# Risk of Short-Term Prostate-Specific Antigen Recurrence and Failure in Patients With Prostate Cancer: A Secondary Analysis of a Randomized Clinical Trial

**DOI:** 10.1001/jamanetworkopen.2023.36390

**Published:** 2023-10-06

**Authors:** Mutlay Sayan, Jiaming Huang, Wanling Xie, Ming-Hui Chen, Marian Loffredo, Elizabeth McMahon, Peter Orio, Paul Nguyen, Anthony V. D’Amico

**Affiliations:** 1 Department of Radiation Oncology, Brigham and Women’s Hospital and Dana Farber Cancer Institute, Boston, Massachusetts; 2 Department of Data Sciences, Dana Farber Cancer Institute, Boston, Massachusetts; 3 Department of Statistics, University of Connecticut, Storrs

## Abstract

**Importance:**

A shorter time interval to prostate-specific antigen (PSA) failure is associated with worse clinical outcomes; however, specific factors defining this state remain unknown.

**Objective:**

To evaluate the factors of a short time interval to PSA failure in order to identify patients for treatment escalation randomized clinical trials.

**Design, Setting, and Participants:**

This secondary analysis of a randomized clinical trial was a secondary analysis of the Dana-Farber Cancer Institute 05-043 trial and included 350 patients with nonmetastatic unfavorable risk prostate cancer (PC).

**Interventions:**

Patients were randomized 1:1 to receive androgen deprivation therapy (ADT) and radiation therapy (RT) plus docetaxel vs ADT and RT.

**Main Outcomes and Measures:**

Cumulative incidence rates curves of PSA failure, defined as PSA nadir plus 2 ng/mL or initiation of salvage therapies, and the Fine and Gray competing risks regression was used to assess the prognostic association between these factors and time to PSA failure.

**Results:**

The study included 350 males who primarily had a good performance status (330 [94.3%] with Eastern Cooperative Oncology Group score of 0), median (range) age of 66 (43-86) years, with 167 (46.6%) having Gleason scores of 8 to 10, and 195 (55.2%) presenting with a baseline PSA of more than 10 ng/mL. After a median (IQR) follow-up of 10.2 (8.0-11.4) years, having a PSA level of 10 ng/mL to 20 ng/mL (subdistribution hazard ratio [sHR], 1.98; 95% CI, 1.28-3.07; *P* = .002) and a Gleason score of 8 to 10 (sHR, 2.55; 95% CI, 1.63-3.99; *P* < .001) were associated with a shorter time to PSA failure, and older age (sHR, 0.82; 95% CI, 0.72-0.93; *P* = .002) was associated with reduced risk for PSA failure after adjusting for other baseline clinical factors. The high-risk category, defined by these 3 factors, was associated with a shorter time to PSA failure (sHR, 2.69; 95% CI, 1.84-3.93; *P* < .001).

**Conclusions and Relevance:**

In this secondary analysis of a randomized clinical trial of males with unfavorable risk PC, young age, PSA of 10 ng/mL or more, and a Gleason score of 8 to 10 estimated a shorter time to PSA failure. A subgroup of males at very high-risk for early PSA failure, as defined by our study, may benefit from treatment escalation with androgen receptor signaling inhibitors or cytotoxic chemotherapy and should be the subject of a prospective randomized clinical trial.

**Trial Registration:**

NCT00116142

## Key Points

**Question:**

What factors are associated with a shorter time interval to prostate-specific antigen (PSA) failure (PSA nadir plus 2 ng/mL or initiation of salvage therapies) in nonmetastatic unfavorable-risk prostate cancer?

**Findings:**

In this secondary analysis of a phase 3 clinical trial with 350 participants, significant factors associated with shorter time to PSA failure were younger than 70 years, a PSA of 10 ng/mL or more, and a Gleason score of 8 to 10. A high-risk group, defined by these 3 factors, had 43.8% risk of PSA failure at 3 years.

**Meaning:**

These findings suggest that males with unfavorable-risk prostate cancer who are at very high-risk for early PSA failure can be identified and may benefit from study in randomized treatment escalation studies.

## Teaser

This secondary analysis of a randomized clinical trial evaluates the factors associated with a short interval to prostate-specific antigen failure among patients with prostate cancer.

## Introduction

Androgen-deprivation therapy (ADT) using a luteinizing hormone-releasing hormone agonist or antagonist is a component of treatment for advanced hormone-sensitive M0 prostate cancer (PC) as several prospective randomized clinical trials have demonstrated that the addition of ADT to radiation therapy (RT) prolongs PC-specific and overall survival (OS).^1,2,3^ However, despite definitive management, up to one third of the patients eventually experience biochemical recurrence.^4^ A current standard of care for patients seeking RT who have a nonmetastatic unfavorable-risk PC is external beam RT and ADT. Combination of ADT with the androgen receptor (AR)–signaling inhibitors, such as abiraterone,^5,6^ enzalutamide,^7^ apalutamide,^8^ and darolutamide,^9,10^ provides additional clinical benefits for males with advanced PC both in the castrate sensitive M1 and castrate resistance M0 disease states. Furthermore, randomized clinical trials demonstrated an OS benefit of docetaxel in males with metastatic castrate-sensitive and -resistant PC.^11,12,13^ However, the addition of docetaxel to RT and ADT in males with nonmetastatic unfavorable-risk PC had negative or inconclusive results.^14,15,16,17^ Thus, there is a need for further study to identify the subgroup of unfavorable-risk PC patients who would benefit from the treatment intensification with AR-signaling inhibitors or cytotoxic chemotherapy.

The prognosis following prostate-specific antigen (PSA) failure in patients with localized PC treated with RT varies widely. In addition to PSA-based end points, such as PSA nadir or PSA doubling time, the shorter time interval from RT to PSA failure has been also found to be associated with worse clinical outcomes (eTable 1 in Supplement 1).^18,19,20,21^ In the Dana-Farber Cancer Institute (DFCI) 95-096 randomized clinical trial comparing RT alone with RT and 6-month ADT, Royce et al^19^ reported that the interval to PSA failure of less than 30 months was a significant surrogate for all-cause mortality. The time to PSA failure of less than 2 years was also linked to worse PC-specific survival in Trans-Tasman Radiation Oncology Group 96.01 trial.^20^ In addition, Dignam et al^21^ reported that biochemical recurrence within 3 years is a surrogate to identify the OS benefits of long-term ADT in the secondary analysis of Radiation Therapy Oncology Group 92-02 trial. Given the clinical significance of the time interval to PSA failure, it is vital to identify the prognostic factors associated with early time recurrence.

With the advent of more advanced treatments, such as AR-signaling inhibitors and docetaxel, an opportunity exists for treatment escalation, yet for whom treatment escalation is most needed remains unknown. In the current study, we present a secondary analysis of DFCI trial 05-043 using individual patient data, evaluating the factors of shorter time interval to PSA failure (ie, within 3 years) to identify patients for treatment escalation randomized clinical trials.

## Methods

This randomized clinical trial was approved by the institutional review board of the Dana-Farber/Harvard Cancer Center and registered with ClinicalTrials.gov.^22^ This study follows the Consolidated Standards of Reporting Trials (CONSORT) reporting guideline. Informed consent was provided by all participants.

### Patient Population and Treatment

Between September 21, 2005, and January 13, 2015, 350 patients from both academic and community-based health centers in the United States, Australia, and New Zealand who has been diagnosed with the 2002 American Joint Commission on Cancer Clinical Stage T1b-T4N0M0 adenocarcinoma of the prostate with at least 1 unfavorable prognostic factor were included in this study. Unfavorable prognostic factors included (1) clinical T2c to T4 or (2) clinical T1b to T2b and 1 of the following: PSA level of more than 10 ng/mL (to convert to ug/L, multiply by 1.0), biopsy Gleason score of 4 + 3 or higher, tertiary grade 5 PC, biopsy Gleason score of 3 + 4 with at least 50% of biopsy cores positive, PSA velocity of more than 2 ng/mL/y, or biopsy or radiographic evidence of seminal vesicle invasion. Additional inclusion criteria included being 30 years or older, having an Eastern Cooperative Oncology Group (ECOG) performance status of 0 or 1, and having adequate hematologic function with a white blood cell count more than 3000/mm^3^, platelet count of more than 105/mm^3^, and a hemoglobin count of more than 8.0 g/dL (to convert to g/L, multiply by 10.0). Furthermore, evidence of metastatic disease was assessed using radionuclide bone scan and computed tomography or magnetic resonance imaging, and males with no evidence of metastasis were considered eligible for enrollment. Participants with pelvic lymph nodes measuring up to 1.5 cm in long axis were permitted. Self-identified race was collected as a baseline characteristic during the enrollment process to assess potential disparities and differences in outcomes.

**Figure 1.**
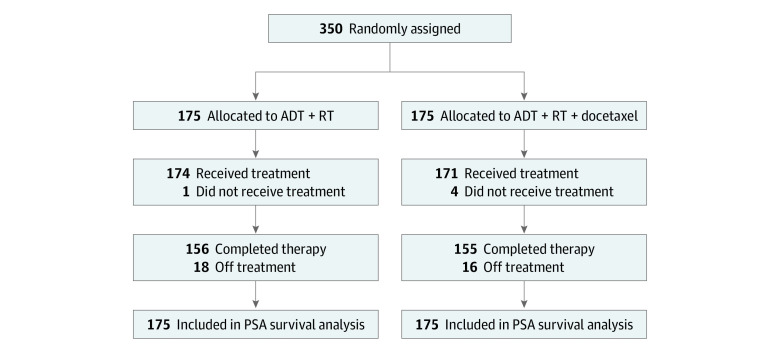
CONSORT Diagram ADT indicates androgen deprivation therapy; RT, radiation therapy.

As shown in Figure 1, the CONSORT diagram, patients were randomly assigned in a 1:1 ratio to receive either 6 months of ADT and RT or 6 months of ADT, RT, and 10 cycles of docetaxel. The PSA survival analysis included a total of 350 patients. All patients received in total 73.7 Gy (after 95% normalization) to the prostate and seminal vesicles using 3-dimensional conformal RT technique or intensity-modulated RT. Pelvic lymph nodes were treated at the discretion of the treating physician. ADT consisted of a luteinizing hormone–releasing hormone agonist and antiandrogen. ADT was given as 2 months of neoadjuvant therapy, 2 months of concurrent therapy with RT, and 2 months of adjuvant therapy. A complete listing of eligibility criteria and treatment specifications is listed in the Trial Protocol in Supplement 2.

### Follow-Up

After the end of treatment, males were followed up with every 6 months for the first 5 years and annually thereafter. A serum PSA was obtained at each follow-up. In addition to routine follow-up assessment, restating with a bone scan and pelvis magnetic resonance imaging or computed tomography were performed at the time of PSA failure. Salvage ADT was recommended when PSA levels rose to approximately 10 ng/mL. All patients were followed from the date of random assignment until death, and the cause of death was centrally reviewed.

### Statistical Analysis

The baseline characteristics of the study cohort at the time of randomization were presented using descriptive statistics in a tabular format. The primary end point was time to PSA failure, defined as time from randomization to earliest date of PSA nadir plus 2 ng/mL or the initiation of salvage therapies, such as ADT, brachytherapy, or radical prostatectomy, or censored at the date of last disease assessment for those alive and without PSA failure. Deaths from non-PC causes were counted as competing risk events.

In this secondary analysis of a randomized clinical trial, the intent of the study was to assess the prognostic association between baseline clinical factors and time to PSA failure. Clinical factors evaluated included age (categorically defined as younger than 60 years vs 60 to 69 years vs 70 years or older), PSA (categorically defined as less than 4 ng/mL vs 4 to less than 10 ng/mL vs 10 to 20 ng/mL vs more than 20 ng/mL), biopsy Gleason score (categorically defined as 8 to 10 vs 7 vs 6 or less), clinical T-category (categorically defined as T3-4 vs T2 vs T1), ECOG performance status (categorically defined as 1 vs 0) and use of pelvic RT. To fulfill this objective, for each baseline factor of interest, graphical cumulative incidence rate (CIR) curves of PSA failure were produced, treating non-PC deaths as competing events. A Gray test was used to compare these curves for each baseline clinical factor of interest. Meanwhile, the CIR estimates of PSA failure at 3 and 5 years were provided with 95% CIs. Furthermore, to more quantitatively evaluate the association of baseline clinical factors of interest with PSA failure, a multivariable competing risk regression for the subdistribution hazard ratio (sHR) using the Fine and Gray model was applied. The model is stratified by regions (US-Northeast, US-West, US-other, Australia, and New Zealand), and randomization arm. Statistical significance was defined as *P* < .05. All analyses were performed using SAS version 9.4 (SAS Institute).

## Results

### Patient Characteristics

The study population included 350 males with a median (range) age of 66 (43-86) years and included 4 Asian males (1.1%), 9 Black males (2.6%), 271 White males (77.4%), and 66 males (18.9%) who selected other as their race. In total, 167 males (46.6%) had a Gleason score of 8 to 10 and 195 (55.2%) with baseline PSA of more than 10 ng/mL. Additionally, 330 males (94.3%) had a good performance status with an ECOG score of 0, as indicated in Table 1. The median (IQR) follow-up was 10.2 (8.0-11.4) years. The overall sample size for the primary analysis included a total of 350 patients. A total of 10 patients withdrew consent or were lost to follow-up during the study period.

**Table 1.** Baseline Clinical Characteristic of 350 Study Patients

Clinical characteristic	Patients, No. (%)
Age, median (range)	66 (43-86)
Race	
Asian	4 (1.1)
Black or African American	9 (2.6)
Other	66 (18.9)
White	271 (77.4)
Ethnicity	
Ethnicity not known	37 (10.6)
Hispanic or Latino	21 (6.0)
Non-Hispanic	292 (83.4)
Year of randomization	
2005-2008	153 (43.7)
2009-2012	168 (48.0)
2013-2015	29 (8.3)
Baseline PSA, ng/mL	
<4	27 (7.7)
4 to <10	128 (36.6)
10 to 20	99 (28.3)
>20	96 (26.9)
ECOG at baseline	
0	330 (94.3)
1	20 (5.7)
Clinical T Stage	
T1c	93 (26.6)
T2a	46 (13.1)
T2b	43 (12.3)
T2c	69 (19.7)
T3a	66 (18.9)
T3b	31 (8.9)
T4	2 (0.6)
Biopsy Gleason score	
3 + 3	9 (2.6)
3 + 4	72 (20.6)
3 + 5	4 (1.1)
4 + 3	102 (29.1)
4 + 4	35 (10.0)
4 + 5	87 (24.9)
5 + 3	3 (0.9)
5 + 4	26 (7.4)
5 + 5	12 (3.4)

Abbreviations: ECOG, Eastern Cooperative Oncology Group; PSA, prostate-specific antigen.

SI conversion factor: To convert PSA to μg/L, multiply by 1.0.

### Factors of Time to PSA Failure

The CIRs of PSA failure at 3 and 5 years by clinical factors are shown in eTable 2 and illustrated in eFigure in Supplement 1. Table 2 displays the result of the Fine and Gray analysis for the factors of time to PSA failure. Factors significantly associated with a shorter time to PSA failure were higher PSA category (PSA 10 ng/mL to 20 ng/mL: sHR, 1.98; 95% CI, 1.28-3.07; *P* = .002; and PSA >20 ng/mL: sHR, 3.44; 95% CI, 2.25-5.26; *P* < .001) and a Gleason score of 8 to 10 (sHR, 2.55; 95% CI, 1.63-3.99; *P* < .001). Older age was associated with reduced risk for PSA failure (sHR, 0.82; 95% CI, 0.72-0.93; *P* = .002).

**Table 2.** Multivariable Competing Risk Regression Estimate of sHR for Biochemical Progression in Fine and Gray’s Model

Parameter	Parameter estimate^a^	sHR (95% CI)	*P* value
Age per 5-y increment^b^	−0.20	0.82 (0.72 to 0.93)	.002
Baseline PSA, ng/mL			
10-20	Reference	1 [Reference]	NA
<4	−0.07	0.93 (0.44 to 1.97)	.86
10-20	0.68	1.98 (1.28 to 3.07)	.002
>20	1.23	3.44 (2.25 to 5.26)	<.001
Biopsy Gleason score			
6 or 3 + 4	Reference		
7 (4 + 3)	0.22	1.25 (0.77 to 2.03)	.37
8-10	0.94	2.55 (1.63 to 3.99)	<.001
Clinical T category			
T1	Reference		
T2	0.38	1.46 (0.91 to 2.35)	.11
T3-4	0.49	1.64 (0.92 to 2.93)	.10
ECOG performance status			
0	Reference		
1	−0.17	0.84 (0.34 to 2.08)	.71
Use of pelvic RT			
No	Reference		
Yes	−0.54	0.58 (0.37 to 0.94)	.03

Abbreviations: ECOG, Eastern Cooperative Oncology Group; RT, PSA, prostate-specific antigen; radiation therapy; sHR, subdistribution hazard ratio.

SI conversion factor: To convert ng/mL to μg/L, multiply by 1.0.

^a^ The multivariable model is also stratified by regions (US-Northeast, US-West, US-Other, Australia, New Zealand) and randomization arm.

^b^ Age was included in the model as a continuous variable; parameter and hazard ratios of age are shown in per 5-year increment.

**Figure 2.**
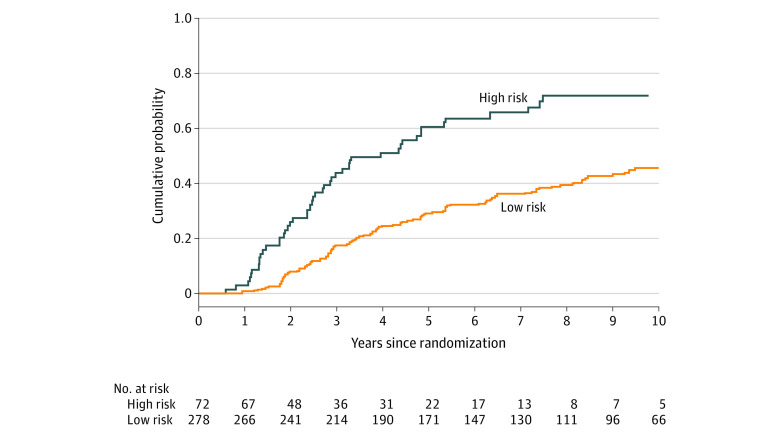
Cumulative Incidence Curves of PSA Failure by Risk Categories High risk category is defined as age younger than 70 years, PSA 10 ng/mL or more, and a Gleason score of 8 to 10.

The 3 significant factors associated with time to PSA failure were used to divide patients into 2 categories. The high-risk category was defined as being younger than 70 years, having a PSA of 10 ng/mL or higher, and having a Gleason score of 8 to 10. Otherwise, it was defined as low risk. Among the males in the high risk category, CIRs of PSA failure at 3 years was 43.8% (95% CI, 31.8%-55.2%) (eTable 2 in Supplement 1). As shown in Figure 2, CIR curves of PSA failure were significantly different between the 2 risk categories (5-year CIR: 60.5% ;95% CI, 47.6%-71.2%; vs 29.0%; 95% CI, 23.6%-34.5%; *P* <.001). In addition to higher clinical T stage and the use of pelvic radiotherapy, the high risk category (sHR, 2.69; 95% CI, 1.84-3.93; *P* < .001) was also associated with a shorter time to PSA failure (Table 3).

**Table 3.** Multivariable Competing Risk Regression Estimate of sHR for Biochemical Progression in Fine and Gray Model

Parameter	Parameter estimate^a^	sHR (95% CI)	*P* value
Risk category			
Low risk	Reference	1 [Reference]	<.001
High risk^b^	0.99	2.69 (1.84 to 3.93)	
Clinical T category			
T1	Reference	1 [Reference]	NA
T2	0.58	1.78 (1.14 to 2.80)	.01
T3-4	0.74	2.09 (1.19 to 3.65)	.01
ECOG performance status			
0	Reference	1 [Reference]	NA
1	−0.12	0.88 (0.35 to 2.26)	.80
Use of pelvic RT			
No	Reference	1 [Reference]	NA
Yes	−0.47	0.62 (0.40 to 0.98)	.04

Abbreviations: ECOG, Eastern Cooperative Oncology Group; RT, radiation therapy; sHR, subdistribution hazard ratio.

^a^ The multivariable model is also stratified by regions (US-Northeast, US-West, US-Other, Australia, New Zealand) and randomization arm.

^b^ The high-risk category was defined as being younger than age 70 years, a prostate-specific antigen of 10 ng/mL or more, and a Gleason score of 8 to 10.

## Discussion

In this study, we examined a cohort of males who experienced PSA failure after ADT and RT with or without docetaxel for nonmetastatic unfavorable-risk PC in the context of a randomized clinical trial. We found 3 significant factors that were associated with a shorter time to PSA failure were being younger than 70 years, having a PSA of 10 ng/mL or more, and having a biopsy Gleason score of 8 to 10. By using these 3 factors, we defined a high-risk group with a 43.8% risk of PSA failure at 3 years. Of note, similar to a previously published randomized clinical trial,^23^ the use of pelvic radiotherapy in our study was associated with a significant reduction in PSA recurrence.

Time to PSA failure was investigated in other retrospective and secondary analyses of males who received RT alone or RT with ADT. In these studies, a shorter time to PSA failure associated with PC-specific mortality,^18^ all-cause mortality,^19^ PC-specific survival,^20^ and OS.^21^ To our knowledge, our study is novel because it is the first analysis combining factors of shorter time to PSA failure. The clinical significance of this study is that this information can be used to select males for a phase 3 study designed to investigate the treatment intensification with AR-signaling inhibitors or cytotoxic chemotherapy.

Combination therapy of ADT and AR-signaling inhibitors leading to greater androgen suppression has been shown to improve clinical outcomes in males with nonmetastatic castrate-resistant PC (nmCRPC). The addition of enzalutamide to ADT was found to prolong OS in males with nmCRPC,^24^ and now the ongoing ENZARAD randomized clinical trial is investigating this combination in males with high-risk PC who are receiving RT.^25^ Long-term data from the phase 3 SPARTAN trial, evaluated apalutamide and ADT vs ADT alone in males with nmCRPC, showed significant improvement in metastasis-free survival (MFS) with the combination.^8^ Similarly, the phase 3 ARAMIS trial revealed that darolutamide plus ADT improved OS compared with ADT alone.^26^ Given the survival benefits observed in males undergoing either apalutamide or darolutamide therapy for nmCRPC and the worse clinical outcomes with a shorter time to PSA failure, one could consider a randomized clinical trial in which the males who were high-risk as defined in our study could be randomized to ADT plus RT with or without apalutamide and/or darolutamide.

Although the addition of docetaxel to ADT is currently not recommended for males with unfavorable-risk PC due to inconclusive results from previous randomized clinical trials,^14,15,16,17,27,28,29^ a subgroup of males as defined by our study may benefit from and should be the subject of future randomized study. The Decipher risk score can also be an alternative strategy to guide treatment escalation for males with unfavorable-risk PC. A recently activated NRG-GU009 trial is now randomizing males with high-risk PC to either intensification or deintensification treatments based on the Decipher risk score.^30^

The use of molecular markers in risk stratification for PC is an active area of research with significant future potential. New tissue-based genomic tests, such as the Decipher, Prolaris, PTEN/TMPRSS2:ERG, Oncotype DX, ConfirmMDx, and ProMark, are being investigated to improve the detection and risk assessment of PC.^31^ Future research will focus on validating these biomarkers and determining their effectiveness in guiding treatment decisions. Furthermore, the integration of molecular markers into clinical practice will require the development of guidelines and algorithms to facilitate their appropriate use in risk stratification and treatment planning. These advancements have the potential to enable more personalized treatment approaches, which is the subject of ongoing NRG-GU009 trial.

### Limitations

This study has limitations. First, it is a secondary subgroup analysis, and therefore these results are hypothesis-generating and should be evaluated in a new cohort study. Second, our observation of the significant factors associated with the shorter time to PSA failure may not be applicable in treatment settings outside the one used in this study. Therefore, the question remains whether these factors are maintained in the postoperative settings. Third, it is important to recognize that the high ECOG performance status observed in this cohort, which was a requirement for inclusion in the initial trial design, may limit the generalizability of our findings to populations with different performance status profiles. Fourth, it is important to acknowledge that the study cohort primarily consisted of non-Hispanic White men, which may limit the generalizability of our findings to more racially diverse populations. Fifth, the patient’s level of comorbidity may affect the survival benefit from the treatment intensification, and therefore future randomized clinical trials should consider prerandomization stratification by comorbidity level in addition to the high-risk group defined by our study.

## Conclusions

In this secondary analysis of a randomized clinical trial of nonmetastatic unfavorable-risk PC, we found that being younger than 70 years, having a PSA of 10 ng/mL or more, and having a Gleason score of 8 to 10 could be used to estimate the shorter time to PSA failure following initial treatment with ADT and RT with or without docetaxel. These results support identifying males at very high risk for early PSA failure who may benefit from treatment escalation and could be studied in the setting of a prospective randomized clinical trial.

## Notes

Supplement 1. **eTable 1.** Time Intervals for PSA Failure in Prostate Cancer Studies **eTable 2.** The Cumulative Incidence Rates (CIR) of PSA Failure at 3 and 5 Years by the Clinical Factors **eFigure.** Cumulative Incidence Curves of PSA Failure by Age Group, PSA Levels, Gleason Score, 23 Clinical T Category, ECOG Performance Score, and Use of Pelvic RT

Supplement 2.
Trial Protocol

Supplement 3.
Data Sharing Statement
