# Supplementary material for: Risk of Short-Term Prostate-Specific Antigen Recurrence and Failure in Patients With Prostate Cancer: A Secondary Analysis of a Randomized Clinical Trial
Source: JAMA Netw Open. 2023 Oct 6;6(10):e2336390. doi: 10.1001/jamanetworkopen.2023.36390 (PMC10559177; doi:10.1001/jamanetworkopen.2023.36390)
Supplement: Supplement 1. — eTable 1. Time Intervals for PSA Failure in Prostate Cancer Studies eTable 2. The Cumulative Incidence Rates (CIR) of PSA Failure at 3 and 5 Years by the Clinical Factors eFigure. Cumulative Incidence Curves of PSA Failure by Age Group, PSA Levels, Gleason Score, 23 Clinical T Category, ECOG Performance Score, and Use of Pelvic RT [file jamanetwopen-e2336390-s001.pdf]

Supplemental Online Content

Sayan M, Huang J, Xie W, et al. Risk of short-term PSA recurrence and failure in prostate cancer patients: a secondary analysis of a randomized clinical trial. *JAMA Netw Open*. 2023;6(10):e2336390. doi:10.1001/jamanetworkopen.2023.36390

eTable 1: Time Intervals for PSA Failure in Prostate Cancer Studies

eTable 2. The Cumulative Incidence Rates (CIR) of PSA Failure at 3 and 5 Years by the Clinical Factors

eFigure. Cumulative Incidence Curves of PSA Failure by Age Group, PSA Levels, Gleason Score, 23 Clinical T Category, ECOG Performance Score, and Use of Pelvic RT

This supplemental material has been provided by the authors to give readers additional information about their work.

© 2023 Sayan M et al. *JAMA Network Open.*

eTable 1: Time Intervals for PSA Failure in Prostate Cancer Studies.

Study	Time Interval for PSA Failure	Clinical Outcome
Buyyounouski et al.[18]	≤ 18 months	Prostate cancer mortality
Royce et al.[19]	< 30 months	All-cause mortality
Denham et al.[20]	< 2 years	Prostate cancer specific survival
Dignam et al.[21]	≤ 3 years	Overall survival

© 2023 Sayan M et al. *JAMA Network Open.*

eTable 2. The cumulative incidence rates (CIR) of PSA failure at 3 and 5 years by the clinical factors

**Covariates of interest**	**No. of events / Total patients**	**CIR at 3 y, % (95% CI)**	**CIR at 5 y, % (95% CI)**
Age group at randomization			
< 60	38/65	31.5 (20.5-43.2)	42.9 (30.4-54.8)
60-69	85/176	21.1 (15.2-27.6)	36.7 (29.3-44.0)
70+	42/109	19.6(12.7-27.7)	29.0 (20.7-37.8)
Baseline PSA (ng/mL)			
< 4	9/27	15.3 (4.6-31.6)	26.8 (11.6-44.8)
4 to <10	45/128	9.6 (5.2-15.6)	21.8 (15.0-29.4)
10-20	50/101	21.4 (13.9-30.0)	35.9 (26.5-45.4)
> 20	61/94	44.8 (34.0-54.9)	57.1 (45.8-67.0)
Biopsy Gleason score			
6 or 3+4	33/81	13.5 (6.9-22.4)	23.4 (14.4-33.7)
7 (4+3)	37/102	15.8 (9.5-23.6)	26.8 (18.6-35.8)
8-10	95/167	31.1 (24.0-38.3)	46.2 (38.3-53.7)
Clinical T category			
T1	33/93	12.5 (6.6-20.3)	25.2 (16.6-34.8)
T2	77/158	25.2 (18.5-32.3)	38.1 (30.3-45.9)
T3-4	55/99	27.8 (19.3-37.0)	40.3 (30.4-49.9)
ECOG performance status			
0	158/330	22.2 (17.8-26.9)	35.3 (30.1-40.6)
1	7/20	28.8 (9.9-51.3)	35.3 (13.5-58.2)
Use of pelvic RT			
No	131/262	24.8 (19.6-30.2)	38.3 (32.3-44.3)
Yes	34/88	15.9 (8.9-24.6)	26.1 (17.0-36.2)
Self-defined risk category[1]			
High risk	47/72	43.8 (31.8-55.2)	60.5 (47.6-71.2)
Low risk	118/278	17.2 (12.9-21.9)	29.0 (23.6-34.5)

[1]High risk category defined as age < 70, PSA ≥ 10 ng/mL and Gleason score in 8-10.
Abbreviations: CIR, cumulative incidence rate; CI, confidence interval.

© 2023 Sayan M et al. *JAMA Network Open.*

eFigure. Cumulative incidence curves of PSA failure by age group (A), PSA levels (B), Gleason score (C), 23 clinical T category (D), ECOG performance score (E) and, use of pelvic RT (F).

(A)

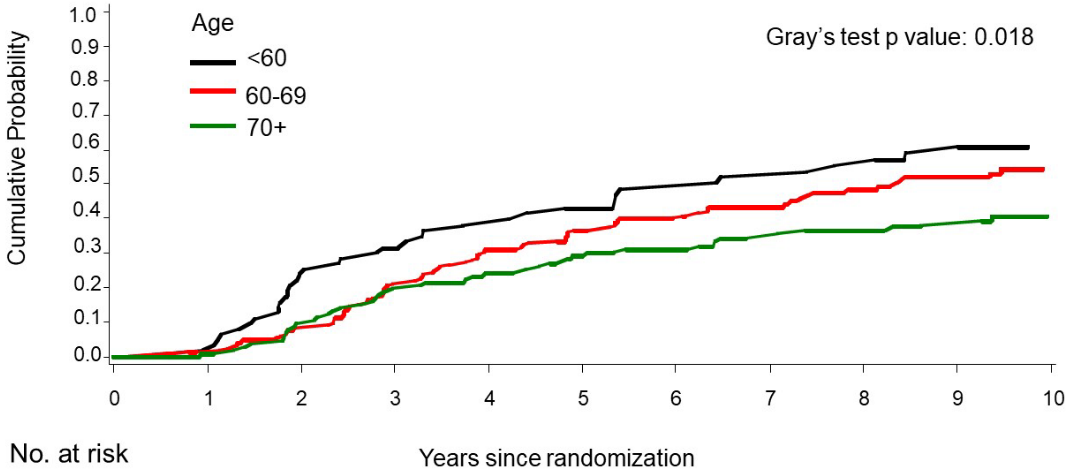

No. at risk

<60	65	63	48	43	38	35	29	27	25	22	15
60-69	176	166	147	124	107	92	78	66	50	42	29
70+	109	104	94	83	76	66	57	50	44	39	27

(B)

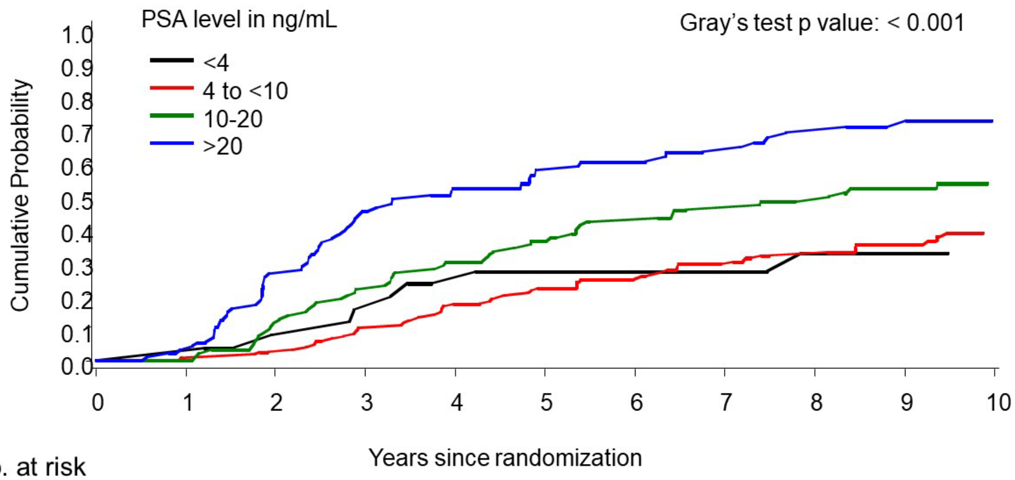

No. at risk

<4	27	27	24	22	19	16	14	14	9	9	5
4 to <10	128	123	119	109	100	93	81	69	61	53	39
10-20	101	98	85	75	66	56	45	40	35	29	22
>20	94	85	61	44	36	28	24	20	14	12	5

© 2023 Sayan M et al. *JAMA Network Open.*

(C)

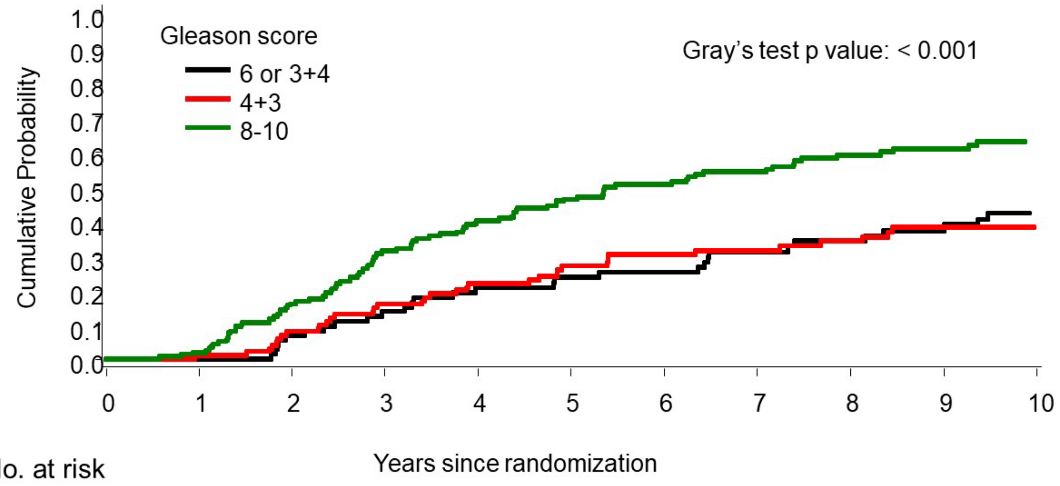

No. at risk

6 or 3+4	81	76	68	62	56	51	49	44	39	37	27
4+3	102	100	90	82	73	65	54	49	43	35	25
8-10	167	157	131	106	92	77	61	50	37	31	19

(D)

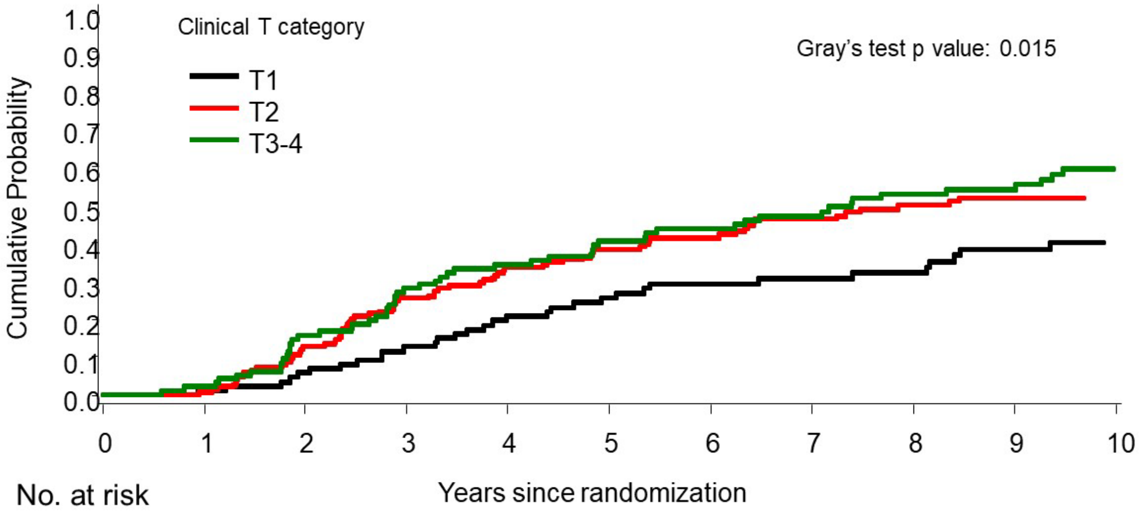

No. at risk

T1	93	87	80	73	66	60	53	46	40	34	29
T2	158	150	127	107	92	80	67	57	46	41	26
T3-4	99	96	82	70	63	53	44	40	33	28	16

© 2023 Sayan M et al. *JAMA Network Open.*

(E)

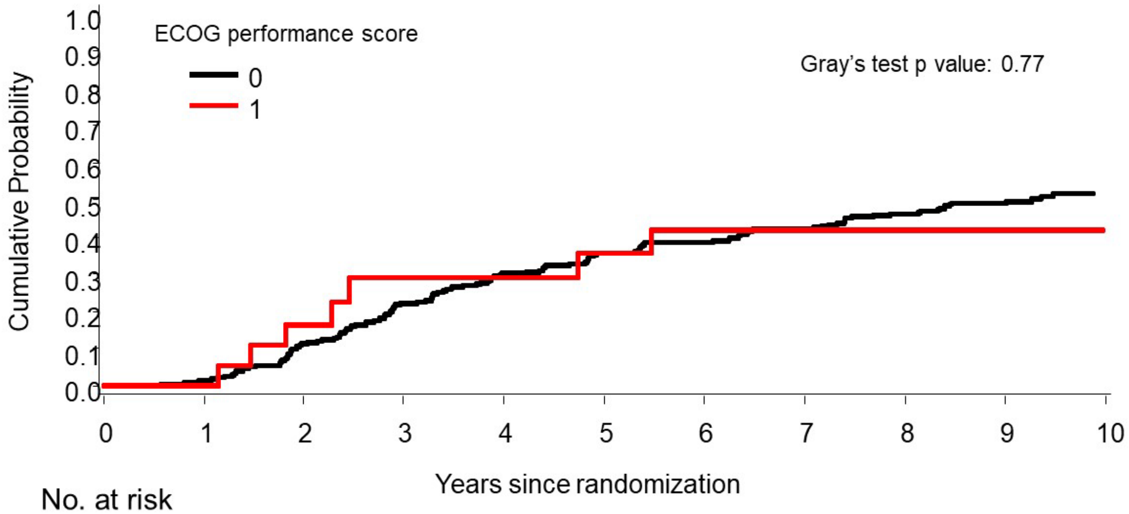

No. at risk

0	330	314	275	239	211	185	158	138	114	99	70
1	20	19	14	11	10	8	6	5	5	4	1

(F)

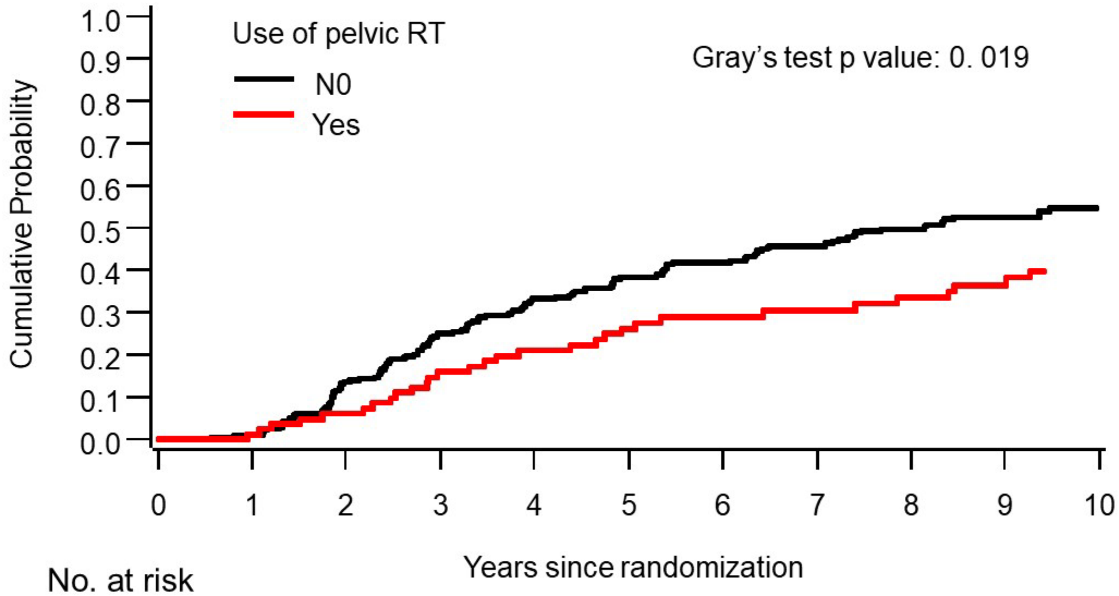

No. at risk

No	262	250	214	185	163	142	120	103	81	69	41
Yes	88	83	75	65	58	51	44	40	38	34	30

© 2023 Sayan M et al. *JAMA Network Open.*
