# Supplementary material for: Risk of Short-Term Prostate-Specific Antigen Recurrence and Failure in Patients With Prostate Cancer: A Secondary Analysis of a Randomized Clinical Trial
Source: JAMA Netw Open. 2023 Oct 6;6(10):e2336390. doi: 10.1001/jamanetworkopen.2023.36390 (PMC10559177; doi:10.1001/jamanetworkopen.2023.36390)
Supplement: Supplement 2. — Trial Protocol [file jamanetwopen-e2336390-s002.pdf]

CLINICAL PROTOCOL

**TITLE:**	**Docetaxel (Taxotere®) plus 6-Month Androgen Suppression and Radiation Therapy vs. 6-Month Androgen Suppression and Radiation Therapy for Patients with High-Risk Localized or Locally Advanced Prostate Cancer: A Randomized Controlled Trial**
**PROTOCOL NUMBER:**	**05-043**
**STUDY DRUG:**	**Taxotere®**
**IND:**	**Non-IND**
**SPONSOR/CRO**	**Dana-Farber Cancer Institute<br>450 Brookline Avenue, Boston, MA 02215-5450<br>Telephone: (617) 732-8821**
**MEDICAL MONITOR/**	**Anthony V. D'Amico, M.D. Ph.D.<br>EMERGENCY CONTACT:<br>Principal Investigator<br>Dana-Farber Cancer Institute<br>450 Brookline Avenue, Boston, MA 02215-5450<br>Telephone: (617) 732- 8821**
PROJECT MANAGER	Marian J. Loffredo, RN OCN<br>Brigham & Women's Hospital<br>Radiation Oncology L-2<br>75 Francis Street<br>Boston, MA 02115<br>Telephone: (617) 732-8821<br>mloffredo@bwh.harvard.edu
**DATE OF PROTOCOL:**	**29 March 2005**
**DATE OF AMENDMENT #1:**	**14 Sept 2005**
**DATE OF AMENDMENT #2:**	**27 Sept 2005**
**DATE OF AMENDMENT #3:**	**22 Nov 2005**
**DATE OF AMENDMENT #4:**	**30 Dec 2005**
**DATE OF AMENDMENT #5:**	**12 May 2006**
**DATE OF AMENDMENT #6:**	**02 Oct 2006**
**DATE OF AMENDMENT #7:**	**12 Feb 2007**
**DATE OF AMENDMENT #8**	**30 March 2007**

DATE OF AMENDMENT #9 1 August 2007

DATE OF AMENDMENT #10: 14, November 2007

DATE OF AMENDMENT # 11: 06, March 2008

DATE OF AMENDMENT #12: 26, June, 2008

DATE OF AMENDMENT #13: 27, January, 2009

DATE OF AMENDMENT #14: 05, June, 2009

DATE OF AMENDMENT #15: 11, August, 2009

DATE OF AMENDMENT #16: 18, DECEMBER, 2009

No Protocol Changes to Amendment # 17

DATE OF AMENDMENT # 18: 20, May, 2010

DATE OF AMENDMENT # 19: 15, February, 2011

DATE OF AMENDMENT #20: 31, October, 2011

DATE OF AMENDMENT #21 31 January 2012

DATE OF AMENDMENT # 22 27, June 2012

DATE OF AMENDMENT # 23 (DFCI# 75) 21, March 2013

DATE OF AMENDMENT # 24 (DFCI# 76) 1 May 2013

DATE OF AMENDMENT #25 (DFCI# 77) 19, JUNE 2013

DATE OF AMENDMENT #26 (DFCI# 86) November 5, 2015

DATE OF AMENDMENT #27 (DFCI#90) August 23, 2016

DATE OF AMENDMENT #28 (DFCI #92) November 14, 2017

DATE OF AMENDMENT #29 (DFCI#94) April 6, 2018

DATE OF AMENDMENT #30 (DFCI#96) August 16, 2019

CONFIDENTIAL INFORMATION

The information contained herein is confidential and the proprietary property of the Dana Farber Cancer Institute and any unauthorized use or disclosure of such information without the prior written authorization of the Dana Farber Cancer Institute is expressly prohibited.

PROTOCOL SIGNATURE PAGE

05-043: Docetaxel (Taxotere®) plus 6-Month Androgen Suppression and Radiation Therapy vs. 6-Month Androgen Suppression and Radiation Therapy for Patients with High-Risk Localized or Locally Advanced Prostate Cancer: A Randomized Controlled Trial

By signing below, the Investigator agrees to adhere to the protocol as outlined and agrees that any changes to the protocol must be approved by Dana Farber Cancer Institute, prior to seeking approval from the Institutional Review Board (IRB) and/or Ethics Review Committee (ERC).

This study will be conducted in accordance with current US Food and Drug Administration (FDA) regulations, good clinical practices (GCPs), the Declaration of Helsinki, and local ethical and legal requirements.

Investigator's Signature: ____
Printed Name: ____
Name of Institution/Company:____
Date: ____

Please submit a signed copy of this page to Dana-Farber Cancer Institute:

Marian J. Loffredo, RN OCN
Project Manager
Brigham & Women's Hospital
Radiation Oncology L-2
75 Francis Street
Boston, MA 02115
617-355-7247
fax: 617-355-6115

TABLE OF CONTENTS **PAGE**

SCHEMA **6**

1.0 INTRODUCTION **7**

2.0 STUDY OBJECTIVES **8**

3.0 PATIENT SELECTION **9**

4.0 PATIENT ENTRY **11**

5.0 TREATMENT PLAN **11**

6.0 REQUIRED DATA **28**

7.0 MODALITY REVIEW **30**

8.0 STATISTICS **31**

9.0 CENTRAL PATHOLOGY REVIEW **25**

REFERENCES **36**

APPENDICES A – C **28-33**

SCHEMA

Patient Selection: Clinical Criteria

Bone Scan (-)
Pelvic Lymph nodes with a long axis of 1.5 cm or less (CT, or MR)

Clinical category T2c, T3a, T3b or T4 as per 2002 AJCC guidelines
-OR-
Clinical category T1b to T2b **AND** any one of the following:

PSA > 10 ng/mL
-OR-
Gleason score ≥ (4+3=7)
-OR-
Any minor tertiary grade of 5
-OR-
Gleason score ≥ (3+4=7) with 50% or more cores positive
-OR-
2.0 ng/ml rise in PSA during the year prior to treatment
or a 2.0 ng/ml average annual rise in PSA based on any PSA values within 3 years prior to treatment
-OR-
Biopsy proven or Radiographic (erMRI) Seminal Vesicle Invasion

Refer to Section 3.0 for a listing of ALL patient eligibility criteria

Randomize

**Group 1**	**Group 2**
Weeks 1 – 9:<br>Total Androgen Suppression#	Weeks 1 – 9:<br>Total Androgen Suppression#<br>+ Docetaxel 60 mg/m^2/q3 weeks x 3 cycles
Weeks 10 – 17:<br>Total Androgen Suppression# + External Beam Radiation Therapy*<br>Pelvic lymph nodes at the discretion of the treating physician	Weeks 10 - 17<br>Total Androgen Suppression# + External Beam Radiation Therapy*<br>Pelvic lymph nodes at the discretion of the treating physician+ Docetaxel 20 mg/m^2/week beginning at week 10 x 7 cycles and continuing through week 16
Weeks 18 – 26:	Weeks 18 - 26<br>Total Androgen Suppression#

Total Androgen Suppression#

Post Therapy Evaluation: (see section 6.0 Required Data).

# Total Androgen Suppression (AST): Casodex (or Bicalutamide) 50 mg. Po q 24°or Eulexin 250 mg. PO q 8 hours with either Eligard - Leuprolide acetate (7.5 mg S.C. q 4 weeks or 22.5 mg. S.C. q 12 weeks) or Lupron 7.5 mg IM q 4 weeks or 22.5 mg IM q 12 weeks) or Zoladex 3.6 mg S.C. q 4 weeks or 10.8 mg S.C. q 12 weeks, approximately 2 months prior to, 2 months concurrent with, and 2 months after completion of radiation therapy. Any LHRH agonist may be used with any dosing schedule (e.g. monthly, 3 month depot, 6 month depot, etc.) as long as the total on study AST treatment does not exceed 26 weeks. As per Section 5.13 patients with up to 4 weeks of AST treatment prior to enrollment will not exceed a total of 30 weeks of AST treatment. Patients being registered from sites outside of the USA are allowed a 2 week leeway if needed.

***External Beam Radiation Therapy: 45 Gy in 25 1.8 Gy doses of radiation therapy will be delivered to the prostate and seminal vesicles using a classic 4-field box technique or any conformal technique including IMRT or protons. An intra-rectal immobilization or localization device may be used. The prostate should be boosted for an additional 25.20 Gy (14 doses of 1.8 Gy) using any conformal technique with up to a 1.5 cm margin from the prostate gland to the block edge. In known cases of seminal vesicle invasion by digital rectal examination (Category T3b) or endorectal MRI, the involved seminal vesicle(s) will also be treated to a total dose of 70.2 Gy.**

Pelvic Lymph Nodes may be treated at the discretion of the physician using the following guidelines.

The superior border of the treatment volume for the prostate and seminal vesicle pelvic fields are at L5/S1 and the pelvic brim should be covered with up to a 2cm margin laterally to include the internal and external iliac nodal chains. The inferior border of the treatment volume of the pelvic field and the posterior border on the treatment volume should have up to a 1.5 cm margin from the prostate and seminal vesicles to the block edge using any conformal technique. The anterior border on the treatment volume should extend to the anterior aspect of the pubic symphysis. The posterior boarded should be no more anterior than S2/3 ensuring at least a 1.5 cm margin posterior aspect of the SV.

When using IMRT only the total prescribed dose of 70.2 Gy to the clinically involved structures is requested. Normal tissue constraints can be applied at the discretion of the treating physician.

1.0 Introduction

The current standard of practice for patients seeking radiotherapeutic management who have a prostate-specific antigen (PSA) level of at least 10 ng/ml or biopsy Gleason score of 7 or higher but clinically localized adenocarcinoma of the prostate is 70 Gray of 3-dimensional conformal external beam radiation (3D CRT) and 6 months of androgen suppression therapy (AST). This practice standard is based on significant improvement in overall survival noted from a randomized study of 70 Gray 3D CRT with or with out 6 months of AST.[1]

The first documented prolongation in median survival for the use of cytotoxic chemotherapy has been reported with the use of Docetaxel for patients with hormone refractory and metastatic prostate cancer.[2-3] Given this benefit in median survival attention has been given to the study of this chemotherapy agent in surgically managed patients with clinically localized high-risk disease. Specifically, the Cancer and Leukemia and Group B is planning a phase III trial of radical prostatectomy with or with out neoadjuvant Docetaxel for these patients. However a randomized trial has not been developed to date for patients with high-risk disease managed primarily with external beam radiation and AST.

Toxicity data from a phase I/II study of concurrent weekly Docetaxel and external beam radiation therapy (EBRT) has been reported.[4] This study has shown that the maximum tolerated dose of Docetaxel with EBRT was 20 mg/m^2 and that the primary acute side effect was diarrhea, which was controlled in all cases with dietary modification and oral agents such as Imodium.

Therefore, the rationale exists for the development of a phase III trial of 70 Gray 3D CRT plus 6 months of AST with or without weekly Docetaxel at 20 mg/m^2.

The rationale for the secondary endpoints listed in 2.2 and 2.3 are:

A PSA DT <3 months has been shown to be a surrogate endpoint amongst men treated with RP or RT for PC but not amongst men treated with RT and 6 mos of ADT+/-Taxotere. If a PSA DT <3 mos is also a surrogate for death from PC in men undergoing RT and 6 mos of ADT +/- Taxotere, it could then be generalized for use in design of future randomized trials building upon the RT + 6 mos ADT or RT and 6 mos ADT + Taxotere paradigms.

Results of RTOG 0521 show improved overall survival (1-side p-value =0.04) to the addition of Taxotere to RT and 2 years of ADT yet this survival benefit appeared to be driven to a large extent by other cause death. We are collecting detailed information on cause of death in DFCI 05-043 (IST 16172) that can enable us to assess the hypothesis as to whether the reduced other cause mortality in the Docetaxel arm is from reduced cardiac death. Given that we know about Taxol coated cardiac stents and a decreased risk of revascularization, Docetaxel, a sister drug to Taxol, may also be cardio protective. [8]

2.0 Study Objectives

Primary Objective:

2.1 To determine if the 5-year estimates of overall survival are increased in patients receiving 6 months of AST, radiation therapy (RT), and 16 weeks of Docetaxel compared to those patients receiving 6 months of AST and radiation therapy in a population of patients known to be at high risk for

progression and death following 6 months of AST and radiation therapy.

Secondary Objective:

2.2 To determine if the proportion of patients with a PSA doubling time of 3 months or more is increased and PSA failure and cancer specific mortality is decreased in patients receiving 6 months of AST, RT, and 16 weeks of Docetaxel compared to those patients receiving 6 months of AST and RT in a population of patients known to be at high risk for progression and death following 6 months of AST and RT.

2.3 To compare the safety profile between the two arms, including AE/SAE, late effect toxicities, and time to onset of cardiovascular events (non-fatal or fatal CVE).

Exploratory Objective:

2.4 To compare overall survival and mortality from prostate cancer, cardiovascular, or other causes by randomized treatment group within subgroups defined by (1) biopsy Gleason score (≤7 versus >7) (2) baseline comorbidity groups (3) baseline serum testosterone levels (Low versus normal).

3.0 Patient Selection

3.1 Eligibility Criteria

3.1.1 Histologic confirmation of prostate cancer[5]

3.1.2 Clinical category T2c, T3a, T3b or T4 as per 2002 AJCC guidelines **-OR-** Clinical category T1b to T2b **AND** any one of the following: PSA > 10 ng/ml **-OR-** Gleason score ≥ (4+3=7) **-OR-** Any minor tertiary grade of 5 **-OR-** Gleason score ≥ (3+4=7) with 50% or more cores positive **-OR-** > 2.0 ng/ml rise in PSA during the year prior to treatment or a 2.0 ng/ml average annual rise in PSA based on any PSA values within 3 years prior to treatment **-OR-** Positive radiographic findings of Seminal Vesicle Invasion (SVI) by endorectal coil MRI or biopsy proven SVI.

PSA rise should be verified after the following criteria have been met:

1) Abstinence from sexual activity/ejaculation 1 week prior
2) No invasive procedures including but not limited to, colonoscopy, erMRI, cystsocopy, prostate needle biopsy, 2 weeks prior.
3) No horseback or bike riding 1 week prior.
4) Genitourinary infection/inflammation ruled out 2 weeks prior.

PSA should be repeated if any of the above conditions apply.

3.1.3 No evidence of metastatic disease (-) bone scan and (-) lymph node assessment (CT, MR) meaning pelvic lymph nodes with a long axis of 1.5 cm or less obtained **within 6 months of study entry and prior to the initiation of hormonal therapy.**

3.1.4 A PSA should be obtained prior to the initiation of hormonal therapy and **within 3 months of study entry**, except when a Transurethral Resection of the Prostate (TURP) has been performed which has falsely lowered the prostate specific antigen (PSA). In this case, the PSA drawn immediately prior to the TURP will be used as the baseline PSA, even if the PSA exceeds 3 months prior to entry.

3.1.5 Adequate hematologic function: WBC > 3,000/ mm^3, platelet count > $10^5/mm^3$, Hemoglobin > 8.0 g/dl obtained **within 1 month of study entry.**

3.16 Creatinine <2.0 mg/dl, Total Bilirubin <ULN (except for Gilbert's syndrome); AST and ALT and Alkaline Phosphatase obtained within 1 month of study entry within the range allowing for eligibility as shown in the table below. In determining eligibility, the value of AST or ALT farthest outside the ULN should be used.

	**AST or ALT:**			
**ALK PHOS:**	**≤ ULN**	**>1x but ≤1.5x**	**>1.5x but ≤5x**	**>5x ULN**
**≤ ULN**	Eligible	Eligible	Ineligible	Ineligible
**>1x but ≤2.5x**	Eligible	Eligible	Ineligible	Ineligible
**>2.5x but ≤5x**	Eligible	Ineligible	Ineligible	Ineligible
**>5x ULN**	Ineligible	Ineligible	Ineligible	Ineligible

3.1.7 ECOG performance status of 0 or 1. (Appendix B) Patients with an ECOG performance status of 1 will not be excluded if wheelchair bound for injury or illness that would not impact his life expectancy.

3.1.8 A free and/or total serum testosterone must be obtained within 3months of patient entry.

3.1.9 Age ≥ 30 year

3.1.10 Peripheral neuropathy: must be ≤ grade 1

3.1.11 Patients must agree to either abstain from sexual intercourse or use a medically acceptable form of contraception while on chemotherapy treatment and for one month after completing chemotherapy treatment

3.1.12 Stratification will occur based on the following 2 factors:

Gleason Grade <=6 to 7 OR 8 to 10 and PSA <=20 or >20 ng/ml (See Schema)

3.2 Exclusion Criteria

3.2.1 Prior history of malignancies that are < 5 years disease free except for any cancers found to be in-situ or would not likely impact a patient's life expectancy with appropriate medical management.

3.2.2 Prior pelvic radiotherapy

3.2.3 Prior hormonal therapy (excluding up to 4 weeks prior to registration/randomization). 5-Alpha Reductase Inhibitors such as Finasteride and Dutasteride to treat Benign Prostatic Hypertrophy are allowed. Patients being registered from sites outside of the USA are allowed a 2 week leeway if needed.

3.2.4 Prior Radical Prostatectomy

3.2.5 Individuals who are unable to tolerate lying still for a 5 - 10 minute radiation treatment because of mental illness or other physical ailment.

3.2.6. Patients with a history of severe hypersensitivity reaction to Docetaxel or other drugs formulated with polysorbate 80.

4.0 Patient Entry

4.1 Confirm eligibility; obtain confirmatory Central Pathology Report from Dr. Renshaw, a requirement for patient registration (See Section 9.0)

4.2 Baseline studies will be obtained (see Section 6.0)

4.3 At the time of patient entry a Patient Registration Form and documents confirming eligibility (PSA/PSA Rise, Gleason sum, clinical stage, (-) bone scan, (-) lymph node by CT or MRI, CBC and differential, LFT's, creatinine, ECOG, free and/or total serum testosterone, original pathology report, central pathology report, and the signed consent form should be faxed to the Project Staff at Dana-Farber Cancer Institute (fax) 617-355-6115 (phone) 617-355-7264 for Evenings or sites outside of the USA (fax) (401)293-5681 Phone (401)-293-5680

5.0 Treatment Plan

5.1 Total Androgen Suppression (AST)

5.1.1 LHRH agonist will consist of either Eligard (Leuprolide Acetate) that will be given SC 7.5 mg q 4 weeks or 22.5 mg every 12 weeks or Lupron that will be given (7.5 mg IM. q 4 weeks or 22.5 mg. IM. q 12weeks) or Zoladex 3.6 mg S.C. q 4 weeks or 10.8 mg S.C. q 12 weeks. These medications will begin 9 weeks prior to the start of radiation therapy (see section 5.3) for a total of 6 injections or 1 injection every 12 weeks for a total of 2 injections, and will continue for a total of 26 weeks. Any LHRH agonist may be used with any dosing schedule (e.g. monthly, 3 month depot, 6 month depot, etc.) as long as the total on study AST treatment does not exceed 26 weeks or 180 days. As per Section 5.13patients with up to 4 weeks of AST treatment prior to enrollment will not exceed a total of 30 weeks of AST treatment. Patients being registered from sites outside of the USA are allowed a 2 week leeway if needed.

5.1.2 Casodex will be administered 50 mg by mouth or Eulexin 250 mg. PO q 8 hours each day starting at least 2 days before the first Eligard, Lupron, or Zoladex injection and will continue for a total of 26 weeks (6 months). No missed or skipped doses will be made up after the 26 weeks of therapy. The generic form of Casodex (Bicalutamide) may be used if available.

Up to 4 weeks of AST with either an LHRH agonist with or without a non-steroidal anti-androgen is permitted prior to patient enrollment as long as all eligibility criteria have been met, including labs and staging. The PSA and radiographic studies (bone scan, CT, MRI) must all be obtained prior to the start of AST and within the designated time frames (PSA within 3 months of study entry and radiographic studies within 6 months of study entry) as outlined in Sections 3.103 and 3.104. This will not be counted as part of the 9 weeks of total AST to be administered prior to the start of radiation therapy as outlined in Section 5.1.1.. Patients being registered from sites outside of the USA are allowed a 2 week leeway if needed.

5.1.3

Hormonal Therapy, per the above guidelines, may be administered by treating physicians at outside facilities as it is part of the standard of care. It is the responsibility of the consenting physician's study team to oversee and ensure that the hormonal therapy is administered according to study guidelines. The consenting physician's study team will also be responsible for entering all hormonal therapy data into the Electronic Data Capture System (eDC).

Time Zero is the date of randomization for men who began up to 4 weeks of AST prior to randomization. Otherwise, Time Zero will be the date of initiation of hormonal therapy (LHRH agonist or anti-androgen, which ever started sooner). It is conceivable and is permitted that due to unforeseen scheduling issues with the patient or physician that up to a 2 week allowance will be permitted without a violation for deviation from the protocol guideline for beginning chemotherapy and hormonal treatment after randomization. Regardless of the definition of Time Zero, the study's primary endpoint will be calculated from the date of randomization

5.1.4 External beam radiation will begin at week 10 (+/- 5 days), and continue for 8 weeks through week 17.

5.2 Chemotherapy (Group 2)

5.2 1 Treatment with Docetaxel at 60mg/m^2 q 3 weeks (1 hour infusion) for 3 cycles will begin with the initiation of the LHRH agonist and be followed by weekly Docetaxel at 20 mg/m^2/week (1 hour infusion) beginning at week 1 of radiation therapy and continuing for 7 weeks. Although the recommended infusion time is 1 hour, this time period may be extended as per institution protocol. If there is disease progression or unacceptable toxicities occur while on treatment, dose modifications may be made but Docetaxel will not exceed the recommended dose and will be completed on week 16.

5.2.2 Patients should receive weekly Docetaxel starting at week 10 or at the start of the first week of radiation therapy, but this may vary +/- 5 days due to uncontrolled circumstances (e.g., holidays or patients' personal conflicts).

5.2.3 Dexamethasone: All patients should undergo pre-medication and post-medication. The following guidelines may be used or the standard of care for your institution may be followed::

The day before Docetaxel infusion: 8 mg p.o. in the morning and 8 mg p.o. in the evening.

The day of Docetaxel infusion: 8 mg p.o. 2 hours before Docetaxel infusion and 8 mg p.o. that same evening.

Note: The dexamethasone dose may be modified at the physician's discretion. The physician may use IV corticosteroid as an alternative route of administration or in addition to by mouth corticosteroids.

The Docetaxel must be administered by an authorized study physician and at an authorized facility that has full IRB approval to enroll and treat patients on this protocol.

5.3 External Beam Radiation Therapy

5.3.1 A PSA will be obtained prior to the start of radiation therapy (and approximately 2 months after the initiation of hormonal therapy).

5.3.2 Megavoltage linear accelerators ($\geq$ 6 MV) with dose rates =/>200 cGy/min will be used..

5.3.3 Diagnostic or treatment planning CT scans can be used to determine the position of the prostate and seminal vesicles.

5.3.4 External beam radiation therapy will commence within 4 weeks (+/- 5 days) after simulation and will not start on a Friday.

5.3.5 45 Gy in 25 1.8 Gy doses of radiation therapy will be delivered to the prostate and seminal vesicles using a classic 4-field box technique or any conformal technique including IMRT or protons. An intra-rectal immobilization or localization device may be used. The prostate should be boosted for an additional 25.20 Gy (14 doses of 1.8 Gy) using any conformal technique with up to a 1.5 cm margin from the prostate gland to the block edge. In known cases of seminal vesicle invasion by digital rectal examination (Category T3b) or endorectal MRI, the involved seminal vesicle(s) will also be treated to a total dose of 70.2 Gy

Pelvic Lymph Nodes may be treated at the discretion of the physician using the following guidelines.

The superior border of the treatment volume for the prostate and seminal vesicle pelvic fields are at L5/S1 and the pelvic brim should be covered with up to a 2cm margin laterally to include the internal and external iliac nodal chains. The inferior border of the treatment volume of the pelvic field and the posterior border on the treatment volume should have up to a 1.5 cm margin from the prostate and seminal vesicles to the block edge using any conformal technique. The anterior border on the treatment volume should extend to the anterior aspect of the pubic symphysis. The posterior boarded should be no more anterior than S2/3 ensuring at least a 1.5 cm margin posterior aspect of the SV.

When using IMRT only the total prescribed dose of 70.2 Gy to the clinically involved structures is requested. Normal tissue constraints can be applied at the discretion of the treating

physician.

5.3.6 Radiation therapy will be delivered once daily (Monday through Friday, holidays and machine maintenance excluded) In the event that a patient needs to miss a radiation treatment due to weather conditions, illness, or personal reason(s), the physician needs to be notified. If the physician approves with the missed day of radiation therapy, a violation will not need to be filed. Any missed days of radiation will be made up at the end of treatment and will be entered into the Electronic Data Capture System (eDC).

5.3.7 Dose should be prescribed to no less than a 95% isodose. If prescribing 70.2 Gy to the 95% isodose line please note that 100% isodose is receiving 73.7 Gy.

5.3.8 Per the physician's discretion, the cone down may be done before or after the prostate and seminal vesicles field. If during the prostate and seminal vesicles radiation a patient needs to stop because of acute radiation induced enteritis, the physician may proceed to the cone down field. Once the cone down course is completed, the physician may decide to reintroduce the prostate and seminal vesicles field if tolerated or complete the radiation using the cone down field.

The following guidelines have been recommended to maximize the radiation therapy dose to the critical tumor containing volume while still adhering to the protocol guidelines and while still protecting normal structures (particularly the rectum):

Prescribe 70.2 Gray in 1.8 Gray fractions with the option to have dosimetry

(a) Keep the Planning Treatment Volume (PTV) minimum dose at 105% of prescription

(b) Place the hot spots (typically 3 to 5%) in the posterior and lateral aspect of the PTV

(c) Suggest to maintain the rectal V70 less than 20% as per QUANTEC guidelines

This enables 8 to 10% more dose than prescription to the critical tumor containing volume (i.e. peripheral zone) of the PTV to a dose range of 75.8 to 77.2 Gray.

External Beam Radiation Therapy, per the above guidelines, may be administered by treating physicians at outside facilities as it is part of the standard of care. It is the responsibility of the consenting physician's study team to oversee and ensure that the radiation therapy is administered according to study guidelines. The consenting physician's study team will also be responsible for entering all radiation therapy data into the Electronic Data Capture System (eDC).

The Radiation Therapy dose should be checked by two individuals to ensure that the prescription is written within the protocol guidelines. These individuals may be from nursing, the physician's group or physics.

5.4 Adverse Events and their Management

5.4.1 Anticipated Toxicity:

5.4.1.1 RADIATION THERAPY:

All patients are seen weekly by their radiation oncologist during radiation therapy. The related morbidity is discussed with the patient using a separate radiation therapy consent form. Common immediate side effects include fatigue, skin redness and irritation in the peri-anal and gluteal folds as well as bowel or bladder irritability. When radiation therapy is delivered in conjunction with Docetaxel chemotherapy, there is an increased risk of diarrhea, which can be successfully managed with dietary modification and oral agents such as Imodium. Late effects (> 6 months following treatment) of radiation therapy include permanent impotence or rectal bleeding. There is a small probability (< 5%) of injury to the bladder, urethra, bowel and other tissues in the pelvis.

5.4.1.2 ANDROGEN SUPPRESSION THERAPY (AST)

The related morbidity is discussed with the patient using a separate medical oncology consent form. Common immediate side effects include fatigue, hot flashes, loss of muscle mass, mild anemia, decreased sexual desire and impotence. Less frequent toxicities are gynecomastia, diarrhea, and pain at the injection site. Cardiovascular or cerebrovascular accidents and skin rashes have rarely been reported.

5.4.1.3 DOCETAXEL

The following adverse events have been identified from clinical trials and/or post-marketing surveillance. Because they are reported from a population of unknown size, precise estimates of frequency cannot be made.

Taxotere has been reported to be associated with diffuse body ache, chest pain, radiation recall phenomenon, some abnormal heart rhythms (atrial fibrillation) and blood clots in the legs or lungs, ECG abnormalities, rapid heart rate, heart attack, skin rashes, allergic reactions, abdominal pain, decreased appetite, constipation, ulcers, esophagitis, inflammation of the colon, infections in the colon, dehydration, bleeding, confusion, conjunctivitis, tearing of the eyes, shortness of breath, fluid in the lungs, pneumonia, kidney malfunction and neuropathy. Very rarely liver inflammation, seizures, fainting, visual disturbances (flashing lights), scarring of the lungs have been reported typically occurring during drug infusion and in association with an allergic reaction. These were reversible upon discontinuation of the infusion.

Hematologic and other toxicity is increased in patients with elevated baseline liver function tests (LFTs) and therefore our eligibility criteria exclude patients with a total serum bilirubin above the upper limit of normal (except in known congenital abnormality) or an AST, ALT, or ALK phos greater than 1.5 times the upper limit of normal.

5.4.2 Toxicity Management

We expect that all side effects associated with radiation therapy, total androgen suppression and Docetaxel to be controllable. The use of routine skin care and a low residue diet will be employed routinely and will temporize most of the acute side effects. Liver function tests will be obtained at the time of study entry for baseline purposes and every 3 weeks thereafter until the 6-month drug course is completed. LFTs should continue to be monitored until they are within normal limits. It will be up to the discretion of the physician to continue obtaining liver function tests after the 6-month drug course is completed.

Patient treatment(s) (including hormonal, radiation or chemotherapy) may be held, discontinued, and/or restarted at the physician's discretion using the recommended dose modifications outlined below when applicable.

Any event requiring a dose modification to any of the aforementioned treatments should be reported on the case report Treatment/Toxicity form and, if applicable, a serious adverse event (see Section 5.4.2.4).

5.4.2.1 LHRH Agonist

Mechanism of action of ***<u>LHRH Agonists</u>***

Eligard (Leuprolide acetate) is a synthetic decapeptide analogue

of Luteinizing Hormone Releasing Hormone (LHRH). It acts as a potent inhibitor of pituitary gonadotropin secretion when administered in the biodegradable formulation. Chronic administration of the drug leads to sustained suppression of the pituitary gonadotropins. Consequently serum levels of testosterone fall to castrate levels 2 - 4 weeks after the initiation of therapy. During routine screening of Eligard, no significant pharmacological activity was apparent in the cardiovascular system, respiratory, central nervous, renal, metabolic, coagulation, or gastric acid secretory systems. The acute toxicity of Eligard has been found to be very low in relation to its pharmacologic potency. Studies have shown that serum levels of testosterone can be reduced and maintained within the castrate level resulting in objective evidence of tumor regression. Other than the occasional transient worsening of cancer symptoms (tumor flare in 1% - 5% of patients) due to an initial temporary rise in testosterone levels on initiating therapy, no significant toxicity apart from that attributed to castration (hot flashes, decreased erections, impotence) has been reported. Reports show that the incidence of localized or generalized rash with patients receiving Eligard is 6%. There have been no reports of bronchospasm in the United States Clinical Trials Program. In general, allergic reactions have been extremely uncommon with Eligard therapy. There have been isolated reports of urethral obstruction, uriticaria, or spinal cord compression. Shortness of breath, cardiac arrhythmia, hyperglycemia, back pain, acute kidney failure, pneumonia, confusion, and weakness were reported in three men. No episodes of anaphylaxis as result of Eligard therapy have occurred in the past.

Lupron and Zoladex have similar toxicities.

5.4.2.2 Casodex

Mechanism of Action of Casodex

Casodex exerts its antiandrogenic action by inhibiting androgen uptake and/or inhibiting nuclear binding of androgen in target tissues such as the prostate.

The reported side effects of Casodex include diarrhea, abdominal cramping or pain, anemia, and mild elevation of liver functions without clinical manifestations, gynecomastia, and decrease in libido and/or impotence. Refer to the package insert for additional information.

Dose Modification Schedule Related to Casodex/Eulexin

If a patient experiences any intolerable side effects, Casodex or Eulexin will be withheld until the side effects resolve. Casodex

may be restarted at 50 mg per day. Alternatively, Casodex may be discontinued at the discretion of the physician. If patient is experiencing gastrointestinal discomfort (cramps, diarrhea), Eulexin will be withheld until the side effects subside and then reintroduce at a tolerated dose not to exceed 750 mg daily.

If a patient experiences a rise in the SGOT or SGPT that is greater than two times the upper limit of normal, then Casodex or Eulexin will be withheld until the liver function tests return to the patient's baseline value or within normal limits. Once normalization occurs Casodex can be restarted at 50 mg per day. Eulexin can be restarted at a decreased dose and increased as tolerated per the physician's discretion. If the liver function tests become elevated again above two times the upper limit of normal then Casodex or Eulexin will be permanently discontinued. Once elevation of the liver function tests has been documented the liver function tests should be obtained every 3 weeks until two consecutive sets of normal LFT's are obtained. Once the 6-month course of hormonal therapy is completed, LFTs will be drawn at the discretion of the treating physician. Skipped or missed doses of Casodex or Eulexin will not be made up.

5.4.2.3 Docetaxel

Mechanism of Action of Docetaxel

Docetaxel is an antineoplastic agent that acts by disrupting the microtubular network in cells that is essential for mitotic and interphase cellular functions. Docetaxel binds to free tubulin and promotes the assembly of tubulin into stable microtubules while simultaneously inhibiting their disassembly. This leads to the production of microtubule bundles without normal function and to the stabilization of microtubules, which results in the inhibition of mitosis in cells. Docetaxel's binding to microtubules does not alter the number of protofilaments in the bound microtubules, a feature that differs from most spindle poisons currently in clinical use.

Dose Modification Schedule Related to Docetaxel

Dose Level Docetaxel	Every 3 week Chemotherapy	Weekly Chemoradiation
Level 0	60mg/m2	20mg/m2
Level -1	54mg/m2	16mg/m2
Level -2	48mg/m2	12mg/m2

Dose adjustments should be based on clinical assessments done on the day before or the day of each treatment cycle. During the every 3 week chemotherapy treatment, the docetaxel will not be escalated once reduced. No matter what dose reductions occur during the every 3 week chemotherapy treatment, once the

patient starts the weekly chemoradiation treatment, the patient should start at 20mg/m2. If the weekly chemoradiation dose is reduced, it will not be re-escalated.
If the patient is unable to tolerate the level -2 dose reduction during the weekly chemoradiation treatments, the docetaxel should be discontinued.

Up to 4 dose reductions are allowed: 2 in the every 3 week chemotherapy and 2 in the weekly chemoradiation therapy. (Excluding Neurotoxicity and Erythema desquamation).

*There are two exceptions to this rule:

Neurotoxicity: For patients who are experiencing neurotoxicity, if the disability persists after 2 reductions, on either the every 3 weeks chemotherapy treatments or on the weekly chemoradiation treatments, all docetaxel should be discontinued. If during the every 3 week chemotherapy treatments the disability resolves to grade 2 or less (after 2 reductions) the patient may continue on the full dose of the weekly chemoradiation treatment.

Erythema, desquamation: For patients who are experiencing erythema, desquamation Grade 3-4: Hold Docetaxel until resolution to < grade 2, and then decrease one dose level for next cycle of therapy. If toxicity resolves to < grade 2 during the every 3 week chemotherapy dose the patient can continue with the full 20 mg/m2 weekly chemoradiation dose as planned . If toxicity recurs, docetaxel should be permanently discontinued.

If the Neurotoxicity and Erythema Desquamation does not resolve within 21 days, the docetaxel should be permanently discontinued.

Preparation and administration of Docetaxel (see *Appendix C)*

5.4.2.3.1 Hematologic toxicities

Weekly blood counts will be obtained throughout Docetaxel treatment including on the day before or the day of each cycle (weeks 1-17). If granulocytes are <1,000/microliter or platelets are <100,000/microliter, hold Docetaxel. If Docetaxel is held, repeat counts weekly and reinstitute therapy when granulocytes > 1,000/µL and platelets > 100,000/µL; if greater than 1 week delay, reinstitute therapy at one lower dose level (i.e., level -1 for first granulocytopenia\thrombocytopenia or level -2 for second episode of granulocytopenia\thrombocytopenia) for Docetaxel.

Dose adjustments for Febrile Neutropenia

For febrile neutropenia on any day of any cycle, defined as ANC < 500 and T > 38.2°C (100.8°F), Docetaxel should be decreased one dose level in all subsequent cycles.

5.4.2.3.2 Hepatic Dysfunction

Dose Modifications for Abnormal Liver Function (Docetaxel)
*Both AST and ALT should be drawn. The more abnormal of the two values (AST or ALT) should be used in determining the dose.

	**AST or ALT:**			
**ALK PHOS:**	**≤ ULN**	**>1x but ≤1.5x**	**>1.5x but ≤5x**	**>5x ULN**
**≤ ULN**	Full Dose	Full Dose	Hold*	Hold*
**>1x but ≤ 2.5x**	Full Dose	Full Dose	Hold*	Hold*
**>2.5x but ≤ 5x**	Full Dose	Reduce Dose	Hold*	Hold*
**>5x ULN**	Hold*	Hold*	Hold*	Hold*

For q3 week Docetaxel therapy or weekly Docetaxel therapy with radiation**:**

*Hold until recovered and then re-treat at a reduced dose (see section 5.4.2.3). "Recovered" is defined as meeting the study baseline eligibility criteria.

<u>Bilirubin</u>: Docetaxel should not be administered to patients with serum total bilirubin >ULN unless they have Gilbert's syndrome as discussed below.. If serum total bilirubin is >ULN on treatment day, hold Docetaxel until serum total bilirubin is ≤ ULN (maximum 21 days), then re-treat at a reduced dose (see section 5.4.2.3). For patients with an elevated bilirubin due to Gilbert's syndrome (and AST, ALT, ALK phos within normal limits), Docetaxel dose modifications will be based solely on AST, ALT and Alkaline Phosphatase values.

5.4.2.3.3 Neurotoxicity

Docetaxel dose modifications for neurotoxicity Grade 3 sensory loss or paresthesia interfering with activities of daily living or Grade 4 permanent sensory loss that interferes with function hold until the toxicity resolves to grade 2 or less and then reinstitute Docetaxel at dose level -1. If during the every 3 week chemotherapy treatments the disability resolves to grade 2 or less (after 2 reductions) the patient may continue on the full dose of the weekly chemoradiation treatment. If disability persists after 2 dose reductions, on either the every 3 weeks chemotherapy treatments or on the weekly chemoradiation treatments, all docetaxel should be discontinued. .

.Up to 2 dose reductions are allowed.

5.4.2.3.4 Gastrointestinal toxicity

For > grade 2 oral ulceration, dysphagia, diarrhea, nausea or vomiting, hold Docetaxel therapy. Once symptoms resolve to grade 1 or less, Docetaxel treatment should be resumed at -1 dose level.

5.4.2.3.5 Fluid retention

There are no dose reductions for fluid retention and treatment for patients with fluid retention is at the discretion of the treating physician. Below are some general guidelines that may be followed:

Patients developing new onset edema, progression of existing edema, or another sign of fluid retention (eg. 2 pound weight gain) are to be treated with oral diuretics. Regimens found to be effective in the management of fluid retention due to Taxotere are listed below.

- Triamterene/hydrochlorothiazide one capsule po qd up to tid.
- Furosemide 40 mg po daily if edema progresses despite Triamterene/hydrochlorothiazide therapy. Potassium supplementation should be given as needed.
- If after a two-week trial, furosemide 40 mg po qd is ineffective, the patient may be treated with furosemide 20 mg po daily plus metolazone 2.5 mg po daily with potassium supplementation as needed.
- Further therapy should be customized depending upon the clinical situation. The clinical tolerance of the patient, the overall tumor response and the medical judgment of the investigator will determine if it is in the patient’s best interest to continue or discontinue treatment.

5.4.2.3.6 Erythema, desquamation

Grade 3-4: Hold Docetaxel until resolution to < grade 2, and then decrease one dose level for next cycle of therapy If toxicity resolves to < grade 2 during the every 3 week chemotherapy dose the patient can continue with the full 20mg/m2 weekly chemoradiation dose as planned . If toxicity recurs, docetaxel should be permanently discontinued. Up to one dose reduction is allowed.

5.4.2.3.7 Hypersensitivity Reactions

Hypersensitivity Reactions

There are no dose reductions for anaphylaxis; patient should be taken off protocol. For all other hypersensitivity reactions, please use the guidelines below or follow the Standard of Care for your institution:

MANAGEMENT OF ACUTE HYPERSENSITIVITY

Severity of Symptoms	Treatment Guidelines
**Mild** symptoms: localized cutaneous reactions such as mild pruritus, flushing, rash	· consider decreasing the rate of infusion until recovery from symptoms, stay at bedside and monitor patient<br>· then, complete Taxotere infusion at the initial planned rate
**Moderate** symptoms: any symptom that is not listed above (mild symptoms) or below (severe symptoms) such as generalized pruritus, flushing, rash, dyspnea, hypotension with systolic BP > 80 mm Hg	· interrupt Taxotere infusion<br>· give diphenhydramine 50 mg IV with or without dexamethasone 10 mg IV; monitor patient until resolution of symptoms<br>· resume Taxotere infusion after recovery of symptoms; depending on the physician's assessment of the patient, Taxotere infusion should be resumed at a slower rate, then increased incrementally to the initial planned rate, *(eg. infuse at an 8 hour rate for 5 minutes, then at a 4-h rate for 5 minutes, then at a 2-h rate for 5 minutes, then finally, resume at the initial planned rate.*<br>· depending on the intensity of the reaction observed, additional oral or IV premedication with an antihistamine should also be given for the **next cycle** of treatment, and the rate of infusion should be decreased initially and then increased back to initial planned rate, *(eg. infuse at an 8 hour rate for 5 minutes, then at a 4-h rate for 5 minutes, then at a 2-h rate for 5 minutes, and finally, administer at the initial planned rate.)*
**Severe** symptoms: any reaction such as bronchospasm, generalized urticaria, systolic BP ≤ 80mm Hg, angioedema	· immediately discontinue Taxotere infusion<br>· give diphenhydramine 50 mg IV with or without dexamethasone 10 mg IV and/or epinephrine as needed; monitor patient until resolution of symptoms.<br>· The same treatment guidelines outlined under moderate symptoms (i.e. the third and fourth bullets) should be followed.
**Anaphylaxis** (NCI grade 4 reaction)	· NO FURTHER STUDY DRUG THERAPY

Patient treatment(s) (including hormonal, radiation or chemotherapy) may be held, discontinued, and/or restarted at the physician's discretion using the recommended dose modifications when applicable.

5.4.2.3.8 Dose Modification for Obese Patients

There is no clearly documented adverse impact of treatment of obese patients when dosing is performed according to actual body weight.

Therefore, all dosing is to be determined solely by the patient's actual weight without any modification unless explicitly described in the protocol. This will eliminate the risk of calculation error and the possible introduction of variability in dose administration. Failure to use actual body weight in the calculation of drug dosages will be considered a major protocol deviation.

5.4.2.4 Adverse Event Reporting:

Definitions:

An **adverse event** (AE) is any untoward medical occurrence in a patient or clinical investigation subject administered a pharmaceutical product and which does not necessarily have a causal relationship with this treatment.

An AE can therefore be any unintended and unfavorable sign (including a clinically significant abnormal laboratory finding, for example), symptom or disease temporally associated with the use of a medicinal product, whether or not considered related to the medicinal product.

An AE **includes**:

- An exacerbation of a pre-existing illness or symptom
- Post-treatment events that occur as a result of protocol-mandated procedures
- A condition detected or diagnosed after study drug administration

An AE **does not include**:

- The disease or disorder being studied or signs and symptoms associated with the disease/disorder unless there is an unexpected worsening of the subject's condition
- A pre-existing disease or condition present at the start of the study that did not worsen
- Elective medical or surgical procedures unless **resulting from** a serious adverse event (see SAE definition below)
- An overdose of the study drug or concurrent medication without any clinical sign or symptom

Adverse events will be recorded from the start of study treatment through completion of study treatment. AEs not resolved at the end of study treatment will be recorded as ongoing.

Please note that adverse events should be documented in the source records of each facility but only Serious Adverse Events will be recorded in the electronic Data Capture System (eDC) after being reported to the DFCI IRB and IRB or Ethics Committee of record

A **serious adverse event** (SAE) is any adverse event occurring during study treatment or within 30 days of the last dose of study drug that:

- results in death
- is life-threatening
- requires inpatient hospitalization or prolongation of existing hospitalization
- results in persistent or significant disability/incapacity
- a congenital anomaly / birth defect

The definition of serious adverse event (experience) also includes *important medical event*. Medical and scientific judgment should be exercised in deciding whether expedited reporting is appropriate in other situations, such as important medical events that may not be immediately life-threatening or result in death or hospitalization but may jeopardize the patient or may require intervention to prevent one of the other outcomes listed in the definition above. These should also usually be considered serious. Examples of such events are intensive treatment in an emergency room or at home for allergic bronchospasm; blood dyscrasias or convulsions that do not result in hospitalization; or development of drug dependency or drug abuse.

In addition, the Dana-Farber Cancer Institute IRB requires SAE reporting of any Grade 2 (moderate) and Grade 3 (serious) Events that are Unexpected and Possibly, Probably or Definitely Related/Asssociated with the Intervention. Grade 4 neutropenia is an expected toxicity from the Docetaxel. Only Grade 4 neutropenia with infection will require reporting as an SAE. Please note that the Common Toxicity Criteria Volume 3.0 (CTCAE) is used for this study.

The definition of "related" is that there is a reasonable possibility that the drug caused the adverse experience.
All SAEs should be immediately documented using the Dana-Farber Cancer Institute (DFCI) SAE Reporting form. Please refer to the Investigator Study Notebook Vol II for the SAE reporting form... The Medical Monitor(s) at the DFCI will determine if a MedWatch 3500A form needs to be filed and will file one if applicable. SAE should be forwarded to:

Anthony V. D'Amico, M.D. Ph.D.
Medical Monitor
Dana-Farber Cancer Institute
450 Brookline Ave.
Boston, MA 02115
Office: 617- 732-8821

Evenings and sites outside of USA

Office: 401-293-5680

Any questions pertaining to serious adverse events (SAEs) should be forwarded to the Project Manager, Marian Loffredo, RN at mloffredo@bwh.harvard.edu or call 617-732-8821.

The Principal Investigator must be informed by the site of an SAE within 24 hours of becoming aware of any SAE that occurs during the course of the clinical study. All serious adverse events must be reported, whether or not they are considered causally related to the study drug. Appropriate clinical, diagnostic, and laboratory measures must be performed to delineate the cause of the SAE in question and the results reported. All tests that reveal an abnormality considered drug related should be repeated at appropriate intervals until:

- The cause is determined
- A return to baseline value occurs
- Stable results over 2 to 3 consecutive readings that are clinically acceptable and safe for the patient

The Principal Investigator is required to assess the causal relationship to the study drug(s) for each SAE to determine if the event is associated with the use of the drug. Events should be classified as associated with the use of the drug if there is a reasonable probability that the experience may have been caused by the drug. **The investigator is also responsible for informing the IRB and/or the Regulatory Authority of the SAE as per local requirements.**

and

Sanofi - Aventis Pharmaceuticals Global Pharmacovigilance and Epidemiology Department either via e-mail: GPEmailbox@sanofi-Aventis.com or (Telefax: 908-231-4827).

and

Attention: Casodex Investigator Sponsored Study (ISS) Safety Representative, AstraZeneca via Telefax: 800-972-4533

5.4.3 Criteria for removal from study:

- Patient decision to withdraw from the study
- Patient noncompliance with the requirements of the protocol
- A patient may be removed from the study if it is believed that the constraints of the protocol are detrimental to the patient health

- Inability to deliver the planned radiation, LHRH agonist or Docetaxel treatment

A patient may be removed for an expected CTC Grade 3 or greater toxicity, any unexpected CTC grade 3 or greater toxicity that is definitely or probably related to study drug, or with disease progression during the first 6 months of treatment.

Common Toxicity Criteria http://ctep.cancer.gov/reporting/ctc.html

6.0 Required Data – please note - source documentation of follow up data including but not limited to physician's notes, lab results and re-staging studies should be scanned to Elizabeth McMahon – emcmahon@lroc.harvard.edu

	Baseline	Weekly during docetaxel infusions (weeks 1-17)	Every 3 weeks	Within 1 week pre radiation therapy, approximately 2 months from start of AST treatment	*Follow up Assessment Every 6 months post radiation therapy for 5 years (+/-90 days), then annually (+/- 90 days) (See below chart)	PSA failure[3] Prior to Salvage Therapy[4] (See Section 7.1)	Progression[3] (See Section 7.2)	Survival (annually +/- 60 days)
Medical History[1]	X				X			X
Physical Exam[1]	X							
Diagnostic prostate biopsy[2]	X							
PSA[3]	X			X	X	X	X	X
Bone Scan[4]	X					X	X	
Lymph node assessment[5]	X							
ECOG performance status	X							
CBC and diff[6]	X	X	X		X[6]			
Liver Function Tests[7]	X		X					
Creatinine	X							
Erectile Function[8]	X							
Free and/or Total Serum Testosterone[9]	X				X	X	X	
Survival[10]					X	X	X	X
Late Effects[11]					X			

Follow up assessments should be done by a Physician, Physician Assistant, Nurse Practitioner, or Nurse. If the patient is unable to be evaluated in person the data may be entered from information obtained via a telephone assessment.

For patients who have progressed within the first five years after randomization, an annual follow may be done. If for any reason a physician decides that the patient may be seen annually rather than every 6 months during the first five years, then a Note-to-File with the reason must be completed. This will be at the discretion of the treating physician.

All efforts to collect and enter the required data in the above chart should continue. However, once patients are more than five years out from treatment, a violation will

not be required for any missing data as long as survival information has been obtained and entered.

Every effort will be made to obtain survival information on all patients. This may include patients who have been previously lost to follow up.

Survival information will be obtained on all patients except for those who specifically requested that no further information be collected.

Second malignancies/new primary malignancies need to be recorded for all patients once identified.

[1] The Medical History and Physical Exam must be completed within 4 weeks prior to or within 2 weeks after study entry and prior to the start of chemotherapy. Assessment and documentation of any of the following should be included in the Medical History at baseline, follow-up, and survival: (These should be recorded if they are present before treatment begins or anytime after (during follow up and survival visits).

Diabetes – If yes IDDM or NIDDM
Hypercholesterolemia
Coronary Artery Disease
Congestive Heart Failure
Peripheral Vascular Disease
Cardiomyopathy
Hypertension
Pulmonary Embolism
Arrhythmias
Heart Attack (MI)
Stroke or TIA

Please include any Medical Management or Surgical Procedures for the treatment of Coronary Artery Disease for example CABG, Stents, Angioplasty and Medications.

[2] A Gleason pattern score of prostate tumor histology is requested. Central Pathology Review of all original prostate biopsy material is required and will be performed by Dr. Andrew Renshaw at the Baptist Hospital of Miami (Section 9.0)

[3] PSAs may be drawn more frequently than the required six month and annual time points during the follow-up period; however, the interim PSA values will not be used to determine PSA failure and/or progression unless there is a significant rise and the physician decides to initiate or add a new salvage therapy. Salvage therapy is defined as medical or surgical hormonal ablation. In these situations the PSA on which the physician based his decision to initiate salvage therapy will be considered the time of PSA failure.

[4] Bone scans will be obtained within 6 months of study entry. Bone scans or PET scans will be obtained for PSA Failure and Progression at the discretion of the treating physician. Record all bone scans and results in the Edc system. .

[5] Lymph node assessment, obtained within 6 months prior to study entry, can be a CT or MRI of the pelvis and is considered negative if the long axis of the pelvic lymph node is 1.5 cm or less.

[6] After completion of the 26 weeks of study treatment, CBCs will be done according to the follow-up schedule of every 6 months for 5 years and then annually thereafter and may be eliminated once they have normalized or returned to patient's baseline or at the discretion of the treating physician.

[7] Liver function tests will be obtained within 1 month prior to study entry for baseline purposes and every 3 weeks after study entry for the 26 weeks of study treatment.

[8] Baseline erectile function should be recorded prior to the initiation of hormone therapy for accurate assessment.

[9] Free and/or total serum testosterone (preferably total) will be obtained at baseline (within 3 months prior to study entry). After radiation therapy +/- 60 days at: 6 months, 18 months, 36 months, at time of PSA failure (see section 7.1), at time of progression (see section 7.2), and when the patient is taken off study (annually) if obtainable.

[10] Survival status is required for all randomized patients regardless of whether they complete the study treatment unless they have withdrawn consent to be followed. For patients who are off study, every effort should be made to obtain annual survival status. If available, enter PSA and Testosterone levels, late effects, all salvage therapies and radiographic findings. This may be obtained via telephone assessment or from documented office visits.

[11] Late effects will be focused on GU/GI including Urinary/Fecal Incontinence; Hematuria; Diarrhea; Rectal Bleeding; and other. Please note if GU/GI late effects are not reported in source documentation, they will be recorded in the Electronic Data Capture as negative. This information may be obtained via telephone assessment.

For baseline studies the following time intervals will be accepted prior to

registration:

1 month: CBC and Diff, LFT's, and Creatinine

3 months: PSA and Free and/or Total Serum Testosterone

6 months: (Lymph node assessment) CT or MRI of the pelvis, bone scan

No time limit: Diagnostic Biopsy

Once a patient is taken "off study" for any reason including additional salvage therapy, long-term follow-up will continue annually to assess PSA, second malignancies and survival.

6.1 Data Collection

For each patient enrolled, an On-Study Visit must be completed in the Electronic Data Capture (eDC) system. This does not apply to those patients who fail screening. For patients who sign consent but fail screening they should be recorded on the Patient Screening Log. For patients who are randomized but do not receive study treatment or are "off study", a Survival Form will be completed annually, unless the patient withdraws consent to be followed. If a patient is withdrawn from the study because of a treatment-limiting adverse event, thorough efforts should be made to clearly document the outcome.

All forms are to be entered electronically via the Electronic Data Capture System (eDC). The data from the On-Study visit should be completed within 2 weeks of patient registration. The data from the Follow-up visit should be submitted within one month of patient visits or assessments.

7.0 Modality Review

7.1 PSA failure

A secondary endpoint of this study is freedom from PSA failure and PSA Doubling Time. PSAs may be drawn more frequently than the required six month or annual time points during the follow-up period; however, the interim PSA values will not be used to determine PSA failure unless there is a significant rise and the physician decides to initiate salvage therapy. Salvage therapy is defined as a medical or surgical hormonal ablation. This may include, but is not limited to, standard marketed antineoplastic therapies including antiandrogens, other hormonal agents, cytotoxic chemotherapy agents, and biologic response modifiers. In these situations the PSA on which the physician based his decision to initiate salvage therapy will be considered the time of PSA failure.

PSA failure is defined as any single PSA value that exceeds the Nadir PSA level + 2 ng/ml. The patient is considered to have sustained PSA failure on the date of that single PSA. At the time of PSA failure a bone scan or PET scan should be obtained at the discretion of the treating physician. (See section 6.0 footnote[4].

Salvage medical or surgical hormonal ablation is recommended to be delivered no later than a PSA level of 10 ng/ml or at the time of a positive bone or PET scan whichever comes first. However, the discussion of salvage therapy is to provide guidance to the investigator regarding treatment at the time of progression; this is not a protocol requirement. Treatment at the time of progression is at the discretion of the physician and patient; however, PSAs are required every 6 months or at annual time points following PSA failure as defined in Section 7.1.

7.2 Progression (2nd PSA failure after salvage therapy)

Progression is two consecutive PSA rises of at least 0.2 ng/ml above the PSA nadir following salvage therapy or once a new salvage therapeutic intervention (including an antiandrogen withdrawal) has been introduced because of concern regarding progression. At the time of progression, the bone scan or PET scan should be obtained every year until the bone scan or PET scan is positive at the discretion of the treating physician. **(See section 6.0 footnote**[4)] Once the bone scan or PET scan is positive subsequent bone scans or PET scans can be obtained at the discretion of the treating physician.

7.3 Distant Failure

The time to distant failure will be defined as the time from randomization until the documentation of a positive bone scan/PET scan or other biopsy proven metastatic disease.

7.4 Survival

The primary end point of this study is overall survival. Prostate cancer-specific survival is a secondary endpoint. Upon the death of a patient, the cause will be determined by the treating physician with all deaths being attributed to prostate cancer if the patient had evidence of a rising PSA on salvage hormonal therapy and/or a previously documented positive bone scan or PET scan. The survival time will be measured from the date of randomization. For patients who are off study, every effort should be made to obtain annual survival status. If available, enter PSA and Testosterone levels, late effects, all salvage therapies and radiographic findings. This may be obtained via telephone assessment or documentation from office visits.

8.0 Statistics

8.1 General Study Design

The primary endpoint of the study is overall survival, defined as time from randomization to death from any cause. Important secondary endpoints include prostate cancer-specific survival and PSA progression-free survival. Toxicity will also be examined. Patients will be stratified for balancing purposes based on Gleason Grade and PSA. Treatments will be assigned using permuted blocks within strata with dynamic balancing within institutions. An intent-to-treat analysis is planned for the study as its primary analysis, including all patients as randomized.

8.2 Sample Size Determination

8.2.1 Original Design

Patients who are randomized to RT + AST are expected to have overall survival at 5 years of 84% based on the results of two randomized studies.[1,7] Assuming an exponential distribution, this is equivalent to median overall survival of 19.9 years. It is hypothesized that patients randomized to RT + AST + Chemotherapy would have overall survival at 5 years of 92%, or median survival of 41.6 years. We assume that the distribution is exponential with a constant failure (hazard) rate and that accrual is uniform during the accrual period. With accrual of 70 eligible patients per year for 5 years and 4 years of additional follow-up, the study will have power of 80% using a one-sided log rank test with significance level of 5%. Full information will be achieved when approximately 53 patients have died.

Two interim analysis of overall survival are planned at 33% (17 deaths) and 66% (35 deaths) of information. The final analysis will be conducted when 53 deaths have been observed. Early stopping for efficacy will be monitored using O'Brien Fleming use function boundaries. The O'Brien Fleming boundary at 33%, 66% and 100% information are 3.2181, 2.1546, and 1.6937 with corresponding one-sided normal significance levels of 0.0006, 0.0156 and 0.0452. Monitoring for early stopping in favor of the null hypothesis (lack of difference in overall survival) will be done using repeated confidence interval methodology similar to that described by Jennison and Turnbull (1989). At each interim analysis a nominal (1-2*alpha) % confidence interval on the hazard ratio will be computed, where alpha is the nominal significance level of the O'Brien Fleming use function boundary (for an overall one-sided 5% level test) at that analysis time. If the confidence interval does not contain the alternative of interest, in particular the target alternative of 92% 5-year survival (hazard ratio of 2.1), then the Data Safety Monitoring Committee may consider stopping the study early for lack of effect.

PSA progression-free survival is defined as the time from randomization to the earliest of PSA failure or death from any cause. PSA failure is defined in Section 7.1 of the protocol. Based on previous studies, [1,7] we assume that patients randomized to RT + AST will have 5-year progression-free rates of 79% and those randomized to RT + AST + Chemotherapy will have 5-year progression-free rates of 89%. Adequate power (88%) for this endpoint using a one-sided 5% test will be available after 5 years of accrual and 4 years of follow-up. Full information will be available when 70 patients have progressed or died. The study does not have statistical power to examine treatment effects within racial and ethnic subgroups; however, these will be described in the study report.

8.2.2 Study Status and Design Change in Feb, 2018

The study completed enrollment in January, 2015, with 350 patients randomized over 9.5 years. Two interim analyses have been conducted at 34% information (18 deaths) and 64% information (34 deaths), respectively, according to the original

design. Full information was reached in August 2017 when 53 patients have died. As of January 2018, 59 deaths were reported.

The study was initially designed with 80% power to detect an hazard ratio of 0.48 (i.e. 5-year OS rate of 0.84 for the AST+RT arm versus 5-year OS rate of 0.92 for the AST+RT+Chemotherapy arm under the exponential distribution) using the one-sided alpha of 0.05. With this amendment, we will extend follow-up until 86 deaths occur, which increases the study power to **90%** based on one-sided alpha level of **0.025**. The new design is based on the actual accrual rate and accounts for two interim analyses that have been conducted. The targeted difference (hazard ratio=0.48) and sample size (N=350) stay the same. The table below provides the expected number of events and associated statistical power with the extended follow-up times.

Design parameters (unchanged):

- RT + AST arm: 5-year OS rate of 0.84 (hazard=0.0349 per year under exponential distribution)
- RT + AST + Chemotherapy arm: 5-year OS rate of 0.92 (hazard=0.0167 per year under exponential distribution)
- Targeted difference: Hazard ratio =0.48
- Total N=350

Design parameters (changed):

- Accrual period: 350 patients were uniformly enrolled over 9.5 years (Sep 2005-Jan 2015)
- Overall Type I error (one-sided): 0.025

Total study duration, years	12(current)	13	14	15	**16**
Expected numbers of deaths	58	65	73	79	**86**
Statistical Power	75.6%	80.5%	84.5%	87.6%	**90.1%**
Approximate calendar times	2018/1	2019/1	2020/1	2021/1	**2022/1**

8.3 Final Analysis and Reporting

Final analysis of overall survival will be performed after the full information (86 deaths) is achieved. Survival distribution will be estimated using the method of Kaplan-Meier; 5 year survival rate and 95% two-sided confidence interval (CI) will be provided by treatment arm. Overall survival between the two arms will be compared using a stratified log-rank test, which includes the stratification factors defined at randomization. A one-sided p-value (from stratified log-rank test) less than 0.024 (or two-sided p-value less than 0.048, accounting for two interim analyses conducted at 21% and 40% information under the new design) will indicate that the experimental arm is superior to the control arm.

Prostate cancer-specific survival will be analyzed using a competing risk model, where cumulative incidence of prostate cancer mortality, cardiovascular (CV) mortality and deaths from other causes will be estimated and compared between treatment arms using Gray's test with adjustment of stratification factors of

randomization.

Analysis of other secondary endpoints, including PSA progression-free survival, time to metastasis, metastasis-free survival will be similar to those described for the OS analysis. A nominal p-value $\leq$ 0.05 (two-sided) is considered as statistically significant.

Time to onset of cardiovascular events (non-fatal or fatal CVE) will be analyzed under a competing risk model. Cumulative incidence function for risk of CVE will be estimated accounting for competing risk of inter-current deaths from non-CV cause. Comparison between treatment groups will be conducted using Gray's test adjusted for baseline stratification factors and history of prior heart/coronary artery disease status.

To compare overall survival and mortality from prostate cancer, cardiovascular, or other causes by randomized treatment group, we will conduct subgroup analysis defined by:

(1) biopsy Gleason score (≤7 versus >7),
(2) baseline comorbidity groups (patients will be classified to "none or minimal" versus "moderate or severe" comorbidity using the established Adult Comorbidity Metric),
(3) baseline serum testosterone levels (Low versus normal).
(4) pre treatment PSA velocity > 2ng/ml/year versus < 2 ng/ml/year

Cox regression and Fine and Gray method will be used to explore whether a significant interaction exists between treatment and subgroup variables on overall mortality and prostate cancer, cardiac, and other-cause mortality, adjusting for the stratification factors defined at randomization. These analyses would be considered exploratory with limited statistical power unless large difference exists between groups regarding treatment effect on these outcomes.

This study will be monitored by the DF/HCC Data and Safety Monitoring Committee (DSMC). The DSMC meets twice each year. For each meeting, all monitored studies are reviewed for safety and progress toward completion. When appropriate, the DSMC will also review interim analyses of outcome data. Safety data may be shared in a blinded fashion (combined across treatment arms) with the study team. Reports regarding outcome will remain blinded to the study team until full information is achieved or as directed by the DSMC.

9.0 Central Pathology Review

9.1 Central pathology review of diagnostic biopsies are required for this study.

9.2 The clinical investigator will mail hematoxylin and eosin (H & E) stained slides, and the pathology report to Dr. Renshaw at the address below An attempt should be made to include all the H&E slides including those that may not show cancer for central review. It is mandatory for all slides to be reviewed if the patient is being randomized using the Gleason =/>3+4=7 with =/> 50% cores positive for eligibility criteria: For sites who are only

allowed to send one slide for central review, the slide that represents the highest Gleason score should be sent.

Andrew Renshaw, MD, Baptist Hospital of Miami, Department of Pathology, 8900 N. Kendall Drive, Miami, Florida 33176

In the event that Dr. Renshaw is on vacation or out of the office for more than one week, the patient may be registered using the institutional review or original pathology results. Dr. Renshaw will review the slides when he returns to the office and his review should be recorded in the Electronic Data Capture System (eDC). If his review is different from the Gleason Score that the patient was randomized with, the project manager will send an e-mail to the study statistician. In addition, a note in the eDC should reflect that the Gleason Score used for randomization if different from the central reviewers.

Questions regarding the pathology review can be directed to Dr. Renshaw at (786-596-6525). Fax # 786-596-5986. All pretreatment biopsies will be assessed for presence of tumor and grading according to Gleason (Appendix A).

References

1. D'Amico AV, Manola J, Loffredo M, Renshaw AA, Della Croce A, Kantoff, Philip. 6-Month Androgen Suppression Plus Radiation Therapy Versus Radiation Therapy Alone For Patients With Clinically Localized Prostate Cancer: A Randomized Controlled Trial. Journal of the American Medical Association 2004; 292:821-7.

2. Eisenberger MA, De Wit R, Berry W, Bodrogi I, Pluzanska A, Chi K, Oudard S, Christine T, James N, Tannock I. A multicenter phase III comparison of Docetaxel (D) + prednisone (P) and mitoxantrone (MTZ) + P in patients with hormone-refractory prostate cancer. Proceedings of the American Society of Clinical Oncology 2004; 23:2 (Abstract # 4).

3. Petrylak DP, Tangen C, Hussain M, Lara PN, Jones J, Taplin ME, Burch P, Greene G, Small E, Crawford ED. SWOG 99-16: Randomized phase III trial of Docetaxel (D)/estramustine (E) versus mitoxantrone(M)/prednisone (p) in men with androgen-independent prostate cancer (AIPCA). Proceedings of the American Society of Clinical Oncology 2004; 23:2 (Abstract # 3).

4. Kumar P, Perrotti M, Weiss R, Todd M, Goodin S, Cummins K, DiPaola RS. Phase I trial of weekly Docetaxel with concurrent three-dimensional conformal radiation therapy in the treatment of unfavorable localized adenocarcinoma of the prostate. J Clin Oncol 2004; 15:1909-15.

5. Gleason DF and the Veterans Administration Cooperative Urological Research Group: Histologic grading and staging of prostatic carcinoma. In: Tannenbaum M, ed. Urologic pathology. Philadelphia, PA: Lea & Febiger; 1977, pp 171-87.

6. Greene FL, Page DL, Fleming ID, Fritz AG, Balch CM, Haller DG, Morrow M. American Joint Committee on Cancer, Manual for staging cancer, 6th edition, New York, NY Philadelphia, Pa.,Springer-Verlag, 2002, p337-46.

7. Bolla M, Gonzalez D, Warde P, Dubois JB, Mirimanoff RO, Storme G, Bernier J, Kuten A, Sternberg C, Gil T, Collette L, Pierart M. Improved survival in patients with locally advanced prostate cancer treated with radiotherapy and goserelin. N Engl J Med. 1997 Jul 31; 337(5):295-300.

Appendix A

PROSTATE

Hospital Name/Address	Patient Name/Information

Type of Specimen ______________________ Histopathologic Type ______________________

Tumor Size ______________________

DEFINITIONS

Primary Tumor (T)

Pathologic			Clinical		
☐	pT2	Organ confined	☐	TX	Primary tumor cannot be assessed
☐	pT2a	Unilateral, one-half of one lobe or less	☐	T0	No evidence of primary tumor
☐	pT2b	Unilateral, involving more than one-half of lobe but not both lobes	☐	T1	Clinically inapparent tumor neither palpable nor visible by imaging
☐	pT2c	Bilateral disease	☐	T1a	Tumor incidental histologic finding in 5% or less of tissue resected
☐	pT3	Extraprostatic extension	☐	Tlb	Tumor incidental histologic finding in more than 5% of tissue resected
☐	pT3a	Extraprostatic extension[2]	☐	T1c	Tumor identified by needle biopsy (e.g., because of elevated PSA)
☐	pT3b	Seminal vesicle invasion	☐	T2	Tumor confined within prostate[3]
☐	pT4	Invasion of bladder, rectum	☐	T2a	Tumor involves one-half of one lobe or less
			☐	T2b	Tumor involves more than one-half of one lobe but not both lobes
			☐	T2c	Tumor involves both lobes
			☐	T3	Tumor extends through the prostate capsule[4]
			☐	T3a	Extracapsular extension (unilateral or bilateral)
			☐	T3b	Tumor invades seminal vesicle(s)
			☐	T4	Tumor is fixed or invades adjacent structures other than seminal vesicles: bladder neck, external sphincter, rectum, levator muscles, and/or pelvic wall

(Pathologic Primary Tumor (T)[1])

Regional Lymph Nodes (N)

Pathologic			Clinical		
☐	pNX	Regional nodes not sampled	☐	NX	Regional lymph nodes were not assessed
☐	pN0	No positive regional nodes	☐	N0	No regional lymph node metastasis
☐	pN1	Metastases in regional node(s)	☐	N1	Metastasis in regional lymph node(s)

Distant Metastasis (M)[5]

Clinical	Pathologic		
☐	☐	MX	Distant metastasis cannot be assessed (not evaluated by any modality)
☐	☐	M0	No distant metastasis
☐	☐	Ml	Distant metastasis
☐	☐	M1a	Non-regional lymph node(s)
☐	☐	Mlb	Bone(s)
☐	☐	Mlc	Other site(s) with or without bone disease.

Biopsy of metastatic site performed........ ☐Y....... ☐N

Source of pathologic metastatic specimen ______________________

Notes

1. There is no pathologic T1 classification.
2. Positive surgical margin should be indicated by an R1 descriptor (residual microscopic disease).
3. Tumor found in one or both lobes by needle biopsy, but not palpable or reliably visible by imaging, is classified as T1c.
4. Invasion into the prostatic apex or into (but not beyond) the prostatic capsule is classified not as T3, but as T2.
5. When more than one site of metastasis is present, the most advanced category is used. pM1c is most advanced.

Appendix B

ECOG PERFORMANCE STATUS*	
**Grade**	**ECOG**
0	Fully active, able to carry on all pre-disease performance without restriction
1	Restricted in physically strenuous activity but ambulatory and able to carry out work of a light or sedentary nature, e.g., light house work, office work
2	Ambulatory and capable of all self-care but unable to carry out any work activities. Up and about more than 50% of waking hours
3	Capable of only limited self care, confined to bed or chair more than 50% of waking hours
4	Completely disabled. Cannot carry on any self-care. Totally confined to bed or chair
5	Dead

Appendix C

PREPARATION AND ADMINISTRATION OF DOCETAXEL

Please note that in June 2013 the 2 vial Taxotere will no longer be available in the USA

Please note that in August, 2013 the 2 vial Taxotere will no longer be available in Australia and New Zealand

Below please find administration and preparation for the one vial Taxotere with correspondence from Sanofi

The 2 vial Taxotere can continue to be used until it has expired

sanofi aventis

Because health matters

Dear Healthcare Professional:

Sanofi-aventis U.S. is informing you about two important changes to the way that Taxotere® (docetaxel) Injection Concentrate vials are being provided for your use:

1. **New single vial formulation:** The new **1-vial Taxotere® at a doubled concentration is now replacing the current 2-vial Taxotere® packaging.** The new 1-vial concentration is 20 mg/mL in comparison to the previous 2-vial preparation, which was 10 mg/mL.

2. **No reconstitution needed:** The new 1-vial Taxotere® **no longer requires reconstitution.** Taxotere® can now be withdrawn from the new 1-vial formulation and injected directly into the IV infusion solution without further dilution.

Administration Precautions
Taxotere® is a cytotoxic anticancer drug and, as with other potentially toxic compounds, caution should be exercised when handling and preparing Taxotere® solutions. The use of gloves is recommended.

If Taxotere® Injection Concentrate, initial diluted solution, or final dilution for infusion should come into contact with the skin, immediately and thoroughly wash with soap and water. If Taxotere® Injection Concentrate, initial diluted solution, or final dilution for infusion should come into contact with mucosa, immediately and thoroughly wash with water.

Contact of the Taxotere® concentrate with plasticized PVC equipment or devices used to prepare solutions for infusion is not recommended. In order to minimize patient exposure to the plasticizer DEHP (di-2-ethylhexyl phthalate), which may be leached from PVC infusion bags or sets, the final Taxotere® dilution for infusion should be stored in bottles (glass, polypropylene) or plastic bags (polypropylene, polyolefin) and administered through polyethylene-lined administration sets.

One-vial TAXOTERE (Injection Concentrate)
TAXOTERE Injection Concentrate requires NO prior dilution with a diluent and is ready to add to the infusion solution.
Please follow the preparation instructions provided below.

Preparation and Administration
DO NOT use the two-vial formulation (Injection Concentrate and diluent) with the one-vial formulation.

One-vial TAXOTERE (Injection Concentrate)
TAXOTERE Injection Concentrate (20 mg/mL) requires NO prior dilution with a diluent and is ready to add to the infusion solution.

1. Taxotere® vials should be stored between 2 and 25°C (36 and 77°F). If the vials are stored under refrigeration, allow the appropriate number of vials of Taxotere® Injection Concentrate vials to stand at room temperature for approximately 5 minutes before use.

2. Aseptically withdraw the required amount of Taxotere® (20 mg docetaxel/mL) with a calibrated syringe and inject into a 250 mL infusion bag or bottle of either 0.9% Sodium Chloride solution or 5% Dextrose solution to produce a final concentration of 0.3 mg/mL to 0.74 mg/mL. If a dose greater than 200 mg of Taxotere® is required, use a larger volume of the infusion vehicle so that a concentration of 0.74 mg/mL Taxotere® is not exceeded

Please see important safety information on pages 3-5 and accompanying full prescribing information, including boxed **WARNING.**

sanofi-aventis U.S., 55 Corporate Drive, PO Box 5925, Bridgewater, NJ 08807-0890
Tel. (908) 981-5000 – www.sanofi-aventis.com

STUDY MEDICATIONS

PREPARATION AND ADMINISTRATION FOR 2 VIAL TAXOTERE

TAXOTERE is a cytotoxic anticancer drug and, as with other potentially toxic compounds, caution should be exercised when handling and preparing TAXOTERE solutions. The use of gloves is recommended. Please refer to **Handling and Disposal** section.

If TAXOTERE Injection Concentrate, initial diluted solution, or final dilution for infusion should come into contact with the skin, immediately and thoroughly wash with soap and water. If TAXOTERE Injection Concentrate, initial diluted solution, or final dilution for infusion should come into contact with mucosa, immediately and thoroughly wash with water.

Contact of the TAXOTERE concentrate with plasticized PVC equipment or devices used to prepare solutions for infusion is not recommended. In order to minimize patient exposure to the plasticizer DEHP (di-2-ethylhexyl phthalate), which may be leached from PVC infusion bags or sets, the final TAXOTERE dilution for infusion should be stored in bottles (glass, polypropylene) or plastic bags (polypropylene, polyolefin) and administered through polyethylene-lined administration sets.

TAXOTERE Injection Concentrate requires two dilutions prior to administration. Please follow the preparation instructions provided below. **Note:** Both the TAXOTERE Injection Concentrate and the diluent vials contain an overfill to compensate for liquid loss during preparation. This overfill ensures that after dilution with the **entire** contents of the accompanying diluent, there is an initial diluted solution containing 10 mg/mL Docetaxel.

The table below provides the fill range of the diluent, the approximate extractable volume of diluent when the entire contents of the diluent vial are withdrawn, and the concentration of the initial diluted solution for TAXOTERE 20 mg and TAXOTERE 80 mg.

Product	Diluent 13% (w/w) ethanol in water for injection Fill Range (mL)	Approximate extractable volume of diluent when entire contents are withdrawn (mL)	Concentration of the initial diluted solution (mg/mL Docetaxel)
Taxotere® 20 mg/0.5 mL	1.88 – 2.08 mL	1.8 mL	10 mg/mL
Taxotere® 80 mg/2 mL	6.96 - 7.70 mL	7.1 mL	10 mg/mL

A. Initial Diluted Solution

1. TAXOTERE vials should be stored between 2 and 25°C (36 and 77°F). If the vials are stored under refrigeration, allow the appropriate number of vials of TAXOTERE Injection Concentrate and diluent (13% ethanol in water for injection) vials to stand at room temperature for approximately 5 minutes.

2. Aseptically withdraw the **entire** contents of the appropriate diluent vial (approximately 1.8 mL for TAXOTERE 20 mg and approximately 7.1 mL for TAXOTERE 80 mg) into a syringe by partially inverting the vial, and transfer it to the appropriate vial of TAXOTERE Injection Concentrate. **If the procedure is followed as described, an initial diluted solution of 10mg docetaxel/mL will result.**

3. Mix the initial diluted solution by repeated inversions for at least 45 seconds to assure full mixture of the concentrate and diluent. Do not shake.

4. The initial diluted TAXOTERE solution (10 mg docetaxel/mL) should be clear; however, there may be some foam on top of the solution due to the polysorbate 80. Allow the solution to stand for a few minutes to allow any foam to dissipate. It is not required that all foam dissipate prior to continuing the preparation process.

5. The initial diluted solution may be used immediately or stored either in the refrigerator or at room temperature for a maximum of 8 hours.

B. Final Dilution for Infusion

1. Aseptically withdraw the required amount of initial diluted TAXOTERE solution (10 mg docetaxel/mL) with a calibrated syringe and inject into a 250 mL infusion bag or bottle of either 0.9% Sodium Chloride solution or 5% Dextrose solution to produce a final concentration of 0.3 to 0.74 mg/mL. If a dose greater than 200 mg of TAXOTERE is required, use a larger volume of the infusion vehicle so that a concentration of 0.74 mg/mL TAXOTERE is not exceeded.

2. Thoroughly mix the infusion by manual rotation.

3. As with all parenteral products, TAXOTERE should be inspected visually for particulate matter or discoloration prior to administration whenever the solution and container permit. If the TAXOTERE initial diluted solution or final dilution for infusion is not clear or appears to have precipitation, these should be discarded.

4. The final TAXOTERE dilution for infusion should be administered intravenously as a 1-hour infusion under ambient room temperature and lighting conditions.

Stability: TAXOTERE infusion solution, if stored between 2 and 25°C (36 and 77°F) is stable for 4 hours. Fully prepared TAXOTERE infusion solution (in either 0.9% Sodium Chloride solution or 5% Dextrose solution) should be used within 4 hours (including the 1 hour i.v. administration).

HOW SUPPLIED

TAXOTERE Injection Concentrate is supplied in a single-dose vial as a sterile, pyrogen-free, non-aqueous, viscous solution with an accompanying sterile, non-pyrogenic, Diluent (13% ethanol in water for injection) vial. The following strengths are available:

TAXOTERE 80 MG/2 ML (NDC 0075-8001-80)

TAXOTERE (Docetaxel) Injection Concentrate 80 mg/2 mL: 80 mg Docetaxel in 2 mL polysorbate 80 and Diluent for TAXOTERE 80 mg (13% (w/w) ethanol in water for injection). Both items are in a blister pack in one carton.

TAXOTERE 20 MG/0.5 ML (NDC 0075-8001-20)

TAXOTERE (Docetaxel)) Injection Concentrate 20 mg/0.5 mL: 20 mg Docetaxel in 0.5 mL polysorbate 80 and diluent for TAXOTERE 20 mg (13% (w/w) ethanol in water for injection). Both items are in a blister pack in one carton.

Storage: Store between 2 and 25°C (36 and 77°F). Retain in the original package to protect from bright light. Freezing does not adversely affect the product.

Handling and Disposal: Procedures for proper handling and disposal of anticancer drugs should be considered. Several guidelines on this subject have been published[1-7]. There is no general agreement that all of the procedures recommended in the guidelines are necessary or appropriate.

REFERENCES

1. OSHA Work-Practice Guidelines for Controlling Occupational Exposure to Hazardous Drugs. *Am J Health-Syst Pharm.* 1996; 53: 1669-1685.

2. American Society of Hospital Pharmacists Technical Assistance Bulletin on Handling Cytotoxic and Hazardous Drugs. *Am J Hosp Pharm.* 1990; 47(95): 1033-1049.

3. AMA Council Report. Guidelines for Handling Parenteral Antineoplastics. *JAMA.* 1985; 253 (11): 1590-1592.

4. Recommendations for the Safe Handling of Parenteral Antineoplastic Drugs. NIH Publication No. 83-2621. For sale by the Superintendent of Documents, US Government Printing Office, Washington, DC 20402.

5. National Study Commission on Cytotoxic Exposure - Recommendations for Handling Cytotoxic Agents. Available from Louis P. Jeffry, Chairman, National Study Commission on Cytotoxic Exposure. Massachusetts College of Pharmacy and Allied Health Sciences, 179 Longwood Avenue, Boston, MA 02115.

6. Clinical Oncological Society of Australia. Guidelines and Recommendations for Safe Handling of Antineoplastic Agents. *Med J Austr.* 1983; 426-428.

7. Jones, RB, et al. Safe Handling of Chemotherapeutic Agents: A Report from the Mt. Sinai Medical Center. *CA-A Cancer Journal for Clinicians.* 1983; Sept/Oct: 258-263.

8. Sandler HM, Rosenthal SA, Sartor O, et al.. A phase III protocol of androgen suppression (AS) and 3DCRT/IMRT versus AS and 3DCRT/IMRT followed by chemotherapy (CT) with docetaxel and prednisone for localized, high-risk prostate cancer (RTOG 0521). 2015 ASCO Annual Meeting J Clin Oncol 33, 2015 (suppl; abstr LBA5002)
