# Supplementary material for: Risk of Short-Term Prostate-Specific Antigen Recurrence and Failure in Patients With Prostate Cancer: A Secondary Analysis of a Randomized Clinical Trial
Source: JAMA Netw Open. 2023 Oct 6;6(10):e2336390. doi: 10.1001/jamanetworkopen.2023.36390 (PMC10559177; doi:10.1001/jamanetworkopen.2023.36390)
Supplement: Supplement 3. — Data Sharing Statement [file jamanetwopen-e2336390-s003.pdf]

Data Sharing Statement

Sayan. Risk of Short-Term Prostate-Specific Antigen Recurrence and Failure in Patients With Prostate Cancer. *JAMA Netw Open*. Published October 06, 2023.
doi:10.1001/jamanetworkopen.2023.36390

Data
Data available: No
